# Obesity Associated Disease in People with Mental Disorders: the Role of Psychotropic Medication and BMI

**DOI:** 10.1007/s10597-025-01536-y

**Published:** 2025-11-11

**Authors:** Mikkel Emil Iwanoff Kolind, Christoph Patrick Beier, Niels-Peter Brøchner Nygaard, Elsebeth Stenager, Claus Bogh Juhl

**Affiliations:** 1 Steno Diabetes Center Odense, Odense University Hospital, Odense, Denmark; 2 Department of Regional Health Research, University of Southern Denmark, Odense, Denmark; 3 Department of Endocrinology, Hospital of South West Jutland, Esbjerg, Denmark; 4 Department of Neurology, Odense University Hospital, Odense, Denmark; 5 Department of Clinical Research, University of Southern Denmark, Odense, Denmark; 6 Department of Neurology, University Hospital of Southern Denmark, Esbjerg, Denmark

**Keywords:** Obesity, Schizophrenia, Depression, Anxiety, Triglycerides, Liver steatosis

## Abstract

Obesity-associated diseases, such as type 2 diabetes, metabolic syndrome, and dyslipidemia, are common in individuals with mental disorders. The relative contribution of mental disorders, psychotropic medication, and BMI remains unclear. In this cross-sectional study, individuals aged 18–60 years with a BMI ≥ 30 kg/m² were referred to the University Hospital of Southern Denmark, Esbjerg, and screened for diabetes, prediabetes, metabolic syndrome, hypertension, blood lipid abnormalities, liver disease, and sleep apnea. Individuals with mental disorders were compared to those without using binomial logistic regression adjusted for BMI, age, sex, and use of psychotropic medication. We included 345 participants with and 317 without mental disorders, all with BMI ≥ 30 kg/m^2^. Participants with mental disorders were younger (40.9 ± 10.9 vs. 44.2 ± 10.8 years) and had higher BMI (42.8 ± 7.9 vs. 40.9 ± 6.4 kg/m²). Adjusted analyses showed no increased odds of obesity-related disease except for blood lipid abnormalities and steatosis. BMI significantly influenced most models but not the association with blood lipid abnormalities. Apart from blood lipid abnormalities and steatosis, obesity-related diseases in individuals with mental disorders appears to be largely attributable to obesity. Weight management should be prioritized, with additional focus on blood lipid abnormalities.

**Supplementary Information:**

The online version contains supplementary material available at 10.1007/s10597-025-01536-y.

## Introduction

The prevalence of obesity has increased over the past decades, contributing significantly to a growing burden of chronic diseases and adverse health outcomes (Collaborators, 2017). Obesity associated disease includes type-2 diabetes (T2D), metabolic syndrome (MetS), blood lipid abnormalities, hypertension, metabolic dysfunction-associated steatotic liver disease (MASLD) and obstructive sleep apnea (OSA). Additionally, there is a higher occurrence of mental disorders (MDs) in people with obesity (Leutner et al., 2023).

The presence of MDs is a risk factor for obesity (Afzal et al., 2021; Holt & Peveler, 2009), however, recent research suggests a bidirectional relationship where obesity often precedes psychiatric disease and may exacerbate existing psychiatric symptoms (Kar & Kar, 2022; Leutner et al., 2023; Sarma et al., 2021). Proposed mechanisms for this interrelationship include adverse effects of antipsychotic and antidepressant medicine, increased stress levels leading to chronic inflammation and inability to maintain a healthy lifestyle (Leutner et al., 2023). Accordingly, people with MDs might be at increased risk of obesity associated somatic diseases. The degree to which this higher risk is due to obesity or MDs per se remains elusive (De Hert et al., 2009; Vancampfort et al., 2015).

The prevalence of T2D is increased in schizophrenia, bipolar disorder, depression, and anxiety disorders (Kremers et al., 2022). Insulin resistance and impaired glucose tolerance may be present at the onset of severe mental illnesses, and has been observed also in treatment-naïve patients with first episode psychosis (Kucukgoncu et al., 2019). Similar metabolic abnormalities are found in relatives of people with schizophrenia, indicating that glucose intolerance may be an intrinsic feature of the disorder, potentially due to shared genetic or environmental risk factors (Fernandez-Egea et al., 2008; Van Welie et al., 2013).

MetS and its key components obesity, hyperglycemia, blood lipid abnormalities and hypertension are all more prevalent in schizophrenia, depression and anxiety (Penninx & Lange, 2018; Sankaranarayanan et al., 2021; van Reedt Dortland et al., 2013). These associations may partly be ascribed to increased obesity rates, lifestyle factors (i.e., low-quality diet, reduced physical activity and disrupted sleep patterns), and psycho-physiological stress (Al-Mughales et al., 2022; van Reedt Dortland et al., 2013). Additionally, the use of psychotropic medications may contribute to both hyperglycemia and blood lipid abnormalities (Pillinger et al., 2020).

In a register study of primary care patients the risk of hypertension was 17%, 11% and 12% higher for schizophrenia, depression and anxiety, respectively, compared to healthy controls, while there was no increased risk among people with bipolar disorder. These differences remained significant when adjusting for the use of psychotropic medication (Pérez-Piñar et al., 2016). The same study found elevated rates of T2D, blood lipid abnormalities, and obesity, as well as an association between antipsychotic use and an increased risk of diabetes, but a reduced risk of hypertension and blood lipid abnormalities, while antidepressant use heightened the risk for all parameters across all MD groups (Pérez-Piñar et al., 2016). Additionally, MDs like depression and anxiety may be associated with altered circadian blood pressure (BP) variability and reduced nocturnal BP dipping (Shahimi et al., 2022).

MASLD is considered the hepatic manifestation of MetS, and its prevalence is significantly higher in people with MDs, where emotional, motivational, and behavioral factors, as well as the effects of psychotropic medications, especially antipsychotics, appear to play a role in disease progression and risk of MASLD (Macavei et al., 2016; Soto-Angona et al., 2020).

The prevalence of MDs is significantly higher among individuals with OSA, with adjusted odds ratios ranging from 1.35 to 2.71 for conditions such as psychosis, bipolar disorder, anxiety, depression, and PTSD, after controlling for age, sex, and ethnicity (Sharafkhaneh et al., 2005).

The contribution of MDs to the risk of obesity-associated diseases remains unclear and is likely driven by a complex interplay of factors. Psychotropic medications appear to play a role in driving this risk, though each disease likely follows its own distinct pathway. This study aims to explore these relationships by examining the presence of obesity-associated disease in people with and without MDs, adjusting for BMI and other confounders, and by evaluating the role of psychotropic medications.

We hypothesized that, compared to healthy participants, (i) participants with MDs have higher odds of obesity-related diseases; (ii) participants with MDs using psychotropic medication have a further increased odds of obesity-related diseases; and (iii) BMI is a key confounder influencing these associations in individuals with and without MDs.

## Methods

This study utilizes baseline data from participants in the South Danish Obesity Initiative at the University Hospital of Southern Denmark, Esbjerg. The initiative provides comprehensive health assessments, designed to screen for obesity-related diseases in people with obesity, with a particular focus on those with coexisting MDs (Juhl et al., 2024). Participants with obesity (BMI ≥ 30 kg/m^2^) aged 18–60 years were referred by general practitioners, local psychiatric treatment units, the Department of Psychiatry, and other departments within the University Hospital of Southern Denmark. This study includes data from participants screened between September 2021 and October 2024.

### Establishing Participant Groups

Participants with reported MDs were assigned to a general MD category (AnyMD). Subgroups consisted of individuals who specifically reported schizophrenia (SCH), bipolar disorder (BD), depression (DEP), or anxiety (ANX). Participants were able to report multiple MDs, resulting in assignment to multiple subgroups. When possible, study nurses validated self-reported diagnoses through patient files and interviews with participants; however, they did not have access to records from general practitioners. Participants were assigned the group without MDs (NoMD) if they met all the following criteria: (i) no reported MD, (ii) not referred from Psychiatric treatment unit, and (iii) no use of any prescription psychotropic medications (Supplementary Information: Table S1). Participants were excluded from group assignment under the following conditions: (i) missing or unclear information regarding MDs (e.g., “don’t know,” “suspects,” or otherwise ambiguous responses without confirmation by the study nurse); (ii) use of psychotropic medication without a reported or confirmed MDs; or (iii) MDs outside the four main diagnostic categories investigated (SCH, BD, DEP, ANX).

### Data Collection Procedures

A study nurse recorded the participants' height, weight, BMI, hip- and waist circumference, assessed body composition via bioimpedance analysis (InBody770^®^; InBody Co., Seoul, Korea), and measured liver stiffness and fat accumulation using transient elastography (FibroScan^®^, Echosens, Paris, France). We evaluated adipose tissue distribution with single-energy CT scans at the umbilicus to assess visceral (VAT) and subcutaneous adipose tissue (SAT).

The nurse measured blood pressure (BP) three times at five-minute intervals, with the Connex Spot Monitor (Welch Allyn, Skaneateles Falls, NY, US) recording the final measurement. Participants wore a 24-hour BP monitor (Cardio Perfect ABPM7100, Welch Allyn, Skaneateles Falls, NY, US) to assess 24-hour BP, daytime and nighttime BP and calculate the nocturnal BP dip.

Fasted blood samples were analyzed for glycated hemoglobin (HbA1c), blood glucose, insulin, total cholesterol (TotalC), low-density lipoprotein cholesterol (LDL-C), high-density lipoprotein cholesterol (HDL-C), triglycerides, alanine aminotransferase, aspartate aminotransferase, and other liver health biomarkers (Supplementary Information: Table S2).

#### Diabetes Screening

Individuals were regarded as having diabetes if they had the diagnosis prior to inclusion or had HbA1c ≥ 48 mmol/mol confirmed by a second test after two weeks. Participants with HbA1c of 42–47 mmol/mol underwent an oral glucose tolerance test and diabetes was confirmed if 2-hour glucose ≥ 11.1 mmol/L. Prediabetes was diagnosed by an HbA1c 42–47 and a normal oral glucose tolerance test.

Insulin resistance was evaluated with the HOMA-IR (Homeostatic Model Assessment of Insulin Resistance). This was calculated using the updated HOMA model (HOMA2) (Wallace et al., 2004) with the HOMA Calculator (Version 2.2.3, Diabetes Trials Unit, University of Oxford, Oxford, U.K. (https://www.rdm.ox.ac.uk/about/our-clinical-facilities-and-units/DTU/software/homa)).

#### Metabolic Syndrome

We defined MetS in accordance with the American Heart Association (Grundy et al., 2005). Participants had to have at least three of the following criteria: Abdominal obesity (waist circumference ≥ 102 cm in men or ≥ 88 cm in women), elevated triglycerides (≥ 1.7 mmol/L or lipid-lowering medication), reduced HDL cholesterol (HDL-C) (< 1.0 mmol/L in men or < 1.3 mmol/L in women or lipid-lowering medication), elevated BP (systolic ≥ 130 mmHg and/or diastolic ≥ 85 mmHg or antihypertensive medication), or elevated fasting glucose (≥ 5.6 mmol/L or antidiabetic medication).

#### Blood Lipid Abnormalities

Blood lipid abnormalities were defined as LDL-C ≥ 3.0 mmol/L, HDL-C < 1.0 mmol/L (men) or < 1.3 mmol/L (women), triglycerides ≥ 1.7 mmol/L, a TotalC-HDL ratio ≥ 6.0 and a triglycerides/HDL-C ratio < 2.97 (men) or < 2.23 (women) (Grundy et al., 2005; Kip et al., 2024; Wakabayashi & Daimon, 2019).

#### Blood Pressure

Hypertension was defined as a daytime systolic/diastolic BP ≥ 135/85 mmHg (Verdecchia et al., 2023). Nocturnal BP variability was assessed using the dip percentage for both systolic and diastolic BP, categorized as: non-dippers (dip% <10%), reverse dippers (dip% <0%), and extreme dippers (dip% >20%).

#### Liver Health Screenings

Liver stiffness measures (LSM) of > 8 KPa were categorizes as intermediate to high and above 12 kPa as high risk of advanced fibrosis (≥ F3) (Berzigotti et al., 2021). We categorized participants with Controlled Attenuation Parameters (CAP) ≥ 270 dB/m as having a high risk of moderate to severe (≥ S2) steatosis (Mertens et al., 2023; Tomaszewski et al., 2021). To further evaluate liver health we calculated Agile scores for all participants. Agile 3+ and Agile 4 are non-invasive composite scoring systems that combines FibroScan results, age, sex, platelet count, and liver enzymes to assess the likelihood of advanced fibrosis (F ≥ 3) and cirrhosis (F4), respectively. We considered Agile 3+ ≥ 0.679 and Agile 4 ≥ 0.565 scores as highly indicative of these conditions (Taru et al., 2024).

#### Sleep Apnea

We screened participants for OSA using the Danish versions of the Berlin Questionnaire (BQ) and the Epworth Sleepiness Scale (ESS). We classified participants as at risk for OSA if they had a BQ score of ≥ 2 or an ESS score of ≥ 15. At-risk participants then underwent further evaluation using a NOX T3 portable respiratory sleep monitor (Nox Medical, Reykjavík, Iceland). We used apnea-hypopnea index (AHI) thresholds of 15 and 30 events per hour to define moderate and severe OSA, respectively (To et al., 2021). Participants already using continuous positive airway pressure (CPAP) therapy were not re-screened and were recorded as having OSA.

### Statistics

We compared demographic characteristics and the prevalence of somatic diseases across all predefined MD groups (AnyMD, SCH, BD, DEP, and ANX), using the NoMD group as the reference. We analyzed continuous variables using Student’s t-tests or Mann-Whitney U tests as appropriate and assessed group distributions with chi-square statistics.

We conducted binomial logistic regression analyses to investigate the odds of obesity-related diseases in individuals with MDs, assess whether the use of psychotropic medications increases these odds, and evaluate the independent impact of BMI on disease outcomes. All models included dichotomized somatic disease categories (e.g., diabetic vs. non-diabetic) as the dependent variable and were adjusted for BMI, age, and sex. Independent variables varied by model type, as described below.

#### Mental Disorder Models

We evaluated the association between MDs and the odds of somatic disease. Independent variables were dichotomized MD groups (e.g., NoMD vs. AnyMD). These models were also used to assess the independent contribution of BMI to somatic disease outcomes.

#### Psychotropic Medication Models

To assess the impact of psychotropic medication use, independent variables were replaced with dichotomized medication use variables (non-users without MDs (the NoMD group) vs. users with MDs (subsample of AnyMD with active prescriptions)) for each medication class (antidepressants, antipsychotics, mood stabilizers, anxiolytics, and hypnotics).

#### Combined Models

For somatic diseases that were significantly associated with the AnyMD group, we further adjusted the models for psychotropic medication use.

We performed all statistical analyses using Stata 18 (StataCorp, College Station, TX, USA). P-values below 0.05 were considered statistically significant. Model results are presented as odds ratios (OR) with 95% Confidence Intervals (CI), expressed as OR [2.5% CI – 97.5% CI].

## Results

As of 25th October 2024, 923 records from participants with a BMI ≥ 30 were available for analysis. A total of 203 participants could not be classified due to missing or ambiguous responses regarding MDs (e.g., ‘don’t know’ or ‘suspects’ without study nurse confirmation). Another 47 were excluded due to psychotropic medication use without a recorded MD, and 11 had MDs outside the four investigated, preventing group assignment.

In total, we included 662 participants (345 with MDs and 317 healthy controls). Among those with MDs, 97 had schizophrenia, 27 had bipolar disorder, 202 had depression, and 246 had anxiety. A total of 317 participants met all criteria for healthy controls (Table 1).

**Table 1:** Participant characteristics and use of psychotropics

	No MD (*n* = 317)	Any MD (*n* = 345)	SCH (*n* = 97)	BD (*n* = 27)	DEP (*n* = 204)	ANX (*n* = 246)
Participant characteristics						
Age^1^	44.2 ± 11.0	39.2 ± 10.9 *	36.5 ± 9.8 *	43.0 ± 9.0	40.3 ± 11.1 *	37.5 ± 10.8 *
BMI^1^	40.9 ± 6.4	42.8 ± 7.9 *	42.2 ± 8.0	41.6 ± 7.2	43.6 ± 8.1 *	43.4 ± 8.0 *
Hip: Waist-ratio^1^	1.06 ± 0.12	1.07 ± 0.11	1.05 ± 0.10	1.10 ± 0.09	1.07 ± 0.10	1.07 ± 0.10
Body Fat^1^ (%)	46.4 ± 6.7	48.1 ± 6.0 *	47.3 ± 6.2	49.0 ± 4.3 *	48.6 ± 6.2 *	48.6 ± 5.9 *
VAT-SAT-ratio^1^	0.44 ± 0.28	0.39 ± 0.24 *	0.40 ± 0.21	0.40 ± 0.24	0.41 ± 0.24	0.38 ± 0.23 *
Sex^2^ (female%)	205 (67.4%)	262 (79.1%) *	66 (70.2%)	26 (96.3%) *	151 (78.6%) *	189 (79.4%) *
In job^2^	268 (85.3%)	133 (38.9%) *	16 (16.5%) *	12 (44.4%) *	78 (38.6%) *	86 (35.3%) *
Any Psychotropic (%)	0 (0.0%)	235 (70.1%)	89 (92.7%)	24 (88.9%)	151 (75.1%)	167 (70.5%)
Anxiolytics (%)	0 (0.0%)	11 (3.2%)	3 (3.1%)	1 (3.7%)	6 (2.9%)	10 (4.1%)
Hypnotics (%)	0 (0.0%)	19 (5.5%)	7 (7.2%)	2 (7.4%)	13 (6.4%)	16 (6.5%)
Mood Stabilizers (%)	0 (0.0%)	68 (19.7%)	26 (26.8%)	16 (59.3%)	48 (23.5%)	47 (19.1%)
Antipsychotics (%)	0 (0.0%)	132 (38.3%)	76 (78.3%)	17 (63.0%)	72 (35.3%)	100 (40.7%)
Antidepressants (%)	0 (0.0%)	147 (42.6%)	39 (40.2%)	14 (51.9%)	117 (57.4%)	106 (43.1%)

Descriptive characteristics of the study population. ^1^metric presented as mean ± SD. ^2^metric presented as count (% prevalence). * indicates a statistically significant difference from No MD group

Abbreviations: *MD *Mental Disorder, *SCH *Schizophrenia, *BD *Bipolar Disorder, *DEP *Depression, *ANX *Anxiety, *VAT-SAT-ratio *Visceral Adipose Tissue to Subcutaneous Adipose Tissue-ratio

### Missing Observations

Cardiorespiratory monitoring was performed only in participants at risk for obstructive sleep apnea (OSA) and excluded those on CPAP treatment, resulting in missing apnea-hypopnea index (AHI) data for 39.7% of the NoMD group and 38.0% of the AnyMD group. Additionally, technical issues led to missing 24-hour blood pressure (BP) data for 45.7% of NoMD and 47.3% of AnyMD participants. As such, the presented hypertension and hypotension rates are based on BP measured during clinical visits. Clinical BP values were similar for participants with and without 24-hour BP data: NoMD group 129.6/84.6 mmHg (available) vs. 129.5/84.1 mmHg (missing); AnyMD group 126.9/83.4 mmHg (available) vs. 124.1/82.4 mmHg (missing), both *p* > 0.05. No other outcome exceeded 5% missingness in either group.

### Participant Characteristics

With the exception of BD, individuals with no reported MDs had lower BMI, higher body fat percentage, higher VAT-SAT-ratio and were younger compared to participants with MDs. Employment rates were significantly lower in participants with MDs compared to participants with no disorders. Use of psychotropics was common in all MD groups (Table 1). The most commonly prescribed medications were antidepressants and antipsychotics, followed by mood stabilizers. Anxiolytics and hypnotics, were consistently taken as adjuncts to other medications except for one participant (Supplementary Information: Fig. S1).

### Unadjusted Somatic Disease Prevalence in Participants with Mental Disorders

Glucometabolic outcomes did not differ significantly between the reference and MD groups. Metabolic syndrome prevalence was elevated in people with depression. Several blood lipid abnormalities were observed in all MD groups across all outcomes, with the exception of LDL-C elevation (Table 2).

**Table 2:** Unadjusted somatic disease prevalence

	No MD (*n* = 317)	Any MD (*n* = 345)	SCH (*n* = 97)	BD (*n* = 27)	DEP (*n* = 204)	ANX (*n* = 246)
**Glucometabolic outcomes**						
IFG	85 (28.3)	90 (27.4)	31 (33.0)	6 (22.2)	58 (30.5)	58 (24.6)
prediabetes	12 (3.8)	9 (2.6)	5 (5.2)	0 (0.0)	5 (2.5)	5 (2.0)
diabetes	36 (11.4)	39 (11.3)	11 (11.3)	2 (7.4)	23 (11.3)	30 (12.2)
HOMA2-IR > 1.4	244 (89.38)	264 (91.35)	75 (92.59)	21 (87.50)	155 (93.94)	194 (92.38)
**Metabolic Syndrome**						
≥ 3 MetS Criteria	227 (71.6)	265 (76.8)	76 (78.4)	17 (63.0)	164 (80.4) *	184 (74.8)
**Blood Lipid outcomes**						
LDL-C ≥ 3 mmol/L	205 (64.7)	219 (63.5)	68 (70.1)	16 (59.3)	131(64.2)	156 (63.4)
triglycerides ≥ 1.7 mmol/L	100 (31.6)	115 (40.6) *	44 (45.4) *	7 (25.9)	97 (47.6) *	79 (39.1) *
HDL < 1.3 (M) or < 1.0 (FM)	151 (49.0)	203 (60.4)*	67 (69.1)*	14 (51.9)	123 (62.8)*	103 (41.9)*
TotalC-HDL Ratio > 6.0	29 (9.2)	49 (14.2)*	19 (19.6)*	2 (7.4)	36 (17.7)*	**35 (14.2)**
Triglycerides-HDL ratio ≥ 2.97 (M) or ≥ 2.23 (FM)	32 (10.6)	73 (22.1)*	27 (28.7)*	4 (14.8)	47 (24.5)*	51 (21.4)*
**Blood pressure outcomes**						
Hypertension	189 (59.6)	172 (49.9)*	35 (36.1)*	12 (44.4)	112 (54.9)	121 (49.2)*
Hypotension	81 (27.7)	101 (29.3)	33 (34.0)	8 (29.6)	51 (29.0)	74 (34.6)
Reverse Dipper >0% BP dip	23 (13.4)	27 (15.8)	10 (25.0)	0 (0.0)	19 (17.3)	21 (16.3)
Extreme Dipper > 20% BP dip	37 (22.2)	23 (13.4)*	5 (12.5)	4 (33.3)	10 (9.1)*	15 (11.6)*
Non-Dipper < 10% BP dip	109 (63.4)	135 (74.2)*	32 (80.0)*	5 (41.7)	87 (79.1)*	97 (75.2)*
**Liver Health outcomes**						
LSM > 8 kPa	63 (19.9)	75 (21.7)	22 (22.7)	5 (18.5)	51 (25.0)	55 (22.4)
LSM > 12 kPa	30 (9.5)	37 (10.7)	10 (10.3)	0 (0.0)	26 (12.8)	27 (11.0)
fibrosis_agile3plus	36 (11.4)	43 (12.5)	13 (13.4)	1 (3.7)	34 (16.7)	30 (12.2)
cirrhosis_agile4	28 (8.8)	39 (11.3)	12 (12.4)	1 (3.7)	31 (15.2) *	26 (10.6)
Steatosis CAP ≥ 270	211 (66.6)	262 (75.9)*	73 (75.3)	17 (63.0)	156 (76.5)*	187 (76.0)*
**Sleep Apnea outcomes**						
AHI ≥ 15 or CPAP treated	133 (57.6)	128 (47.6)*	30 (47.6)	13 (59.1)	82 (50.0)	91 (47.2)*
AHI ≥ 30	40 (20.9)	23 (10.8)*	8 (16.7)	2 (9.5)	15 (11.7)*	20 (13.3)

Prevalence of obesity-associated disease outcomes among individuals with different mental disorders compared to a group without mental disorders. Significant associations (*p* < 0.05) based on chi-square tests are indicated with an asterisk (*)

Abbreviations: *MD *Mental Disorder, *SCH *Schizophrenia, *BD *Bipolar Disorder, *DEP *Depression, *ANX *Anxiety, *IFG *Impaired Fasting Glucose, *HOMA2-IR *Homeostasis Model Assessment of Insulin Resistance, *MetS *Metabolic Syndrome, *LDL-C *Low-Density Lipoprotein Cholesterol, *HDL* High-Density Lipoprotein Cholesterol, *LSM *Liver Stiffness Measurement, *CAP *Controlled Attenuation Parameter, *AHI *Apnea-Hypopnea Index, *CPAP *Continuous Positive Airway Pressure

Compared to NoMD, rates of hypertension tended to be lower across MD groups. Nocturnal BP dipping was less pronounced in MD groups than NoMD. While there was no difference in reverse dipping, the frequency of non-dipping was higher and extreme dipping lower for MD groups compared to NoMD (Table 2).

Most liver health outcomes were comparable across groups except for an elevated frequency of cirrhosis based on Agile 4 score in the DEP group and an increased rate of elevated steatosis risk in the AnyMD, DEP and ANX groups (Table 2).

Rates of OSA tended to be lower in MD groups compared to NoMD (Table 2).

### Adjusted Odds of Obesity Associated Disease Outcomes

After adjusting for BMI, age, and sex, the OR of impaired fasting glucose (IFG) were significantly higher in SCH (OR = 1.9 [1.1–3.4]) compared to NoMD. The odds of metabolic syndrome (MetS) were significantly increased in AnyMD (OR = 1.6 [1.1–2.3]) and DEP (OR = 1.8 [1.1–2.8]) compared to NoMD (Table 3).

**Table 3:** Adjusted odds ratios of obesity associated disease outcomes for people with mental disorders compared to participants without mental disorders

	Any MD OR (95% CI)	SCH OR (95% CI)	BD OR (95% CI)	DEP OR (CI95%)	ANX OR (95% CI)
Glucometabolic Health					
IFG	1.2 (0.8–1.8)	1.9 (1.1–3.4)*	1.0 (0.4–2.8)	1.3 (0.8–2.0)	1.1 (0.7–1.7)
Prediabetes	0.7 (0.3–1.8)	1.7 (0.5–5.5)	0.7 (0.2–3.3)	0.6 (0.2–1.9)	0.6 (0.2–1.9)
Diabetes	1.2 (0.7–2.1)	1.4 (0.6–3.0)	0.7 (0.3–2.7)	1.1 (0.6–2.0)	1.5 (0.8–2.7)
HOMA2-IR > 1.4	1.1 (0.6–2.1)	0.7 (0.2–2.4)	1.1 (0.3–4.1)	1.5 (0.7–3.3)	0.9 (0.3–2.3)
HOMA2-Beta% >50	3.2 (0.8–13.0)	7.2 (1.2–42.1)*	5.7 (0.5–71.1)	1.5 (0.2–9.5)	4.40 (1.0–19.3)*
**Metabolic Syndrome**					
≥ 3 MetS Criteria	1.6 (1.1–2.2)*	1.2 (0.4–3.1)	0.9 (0.4–2.0)	1.8 (1.1–2.8)*	1.3 (0.6–2.6)
**Blood Lipids**					
LDL-C ≥ 3 mmol/l	0.9 (0.7–1.3)	1.4 (0.8–2.3)	0.6 (0.3–1.4)	1.0 (0.7–1.4)	0.9 (0.6–1.3)
Triglycerides ≥ 1.7 mmol/l	2.0 (1.4–2.8)*	2.1 (1.3–3.4)*	1.0 (0.4–2.5)	2.4 (1.6–3.6)*	1.9 (1.3–2.9)*
HDL-C < 1.3 (M) or < 1.0 (FM)	1.2 (0.8–1.6)	1.7 (1.0–2.8)*	0.9 (0.4–2.0)	1.4 (0.9–2.1)	1.3 (0.9–1.9)
TotalC-HDL Ratio > 6.0	1.9 (1.1–3.1)*	2.4 (1.2–4.7)*	1.1 (0.2–4.9)	2.4 (1.4–4.2)*	1.8 (1.0–3.1)
Triglycerides-HDL ratio ≥ 2.97 (M) or ≥ 2.23 (FM)	2.6 (1.6–4.1)*	3.5 (1.9–6.4)*	1.9 (0.6–6.1)	3.1 (1.8–5.2)*	2.4 (1.4–4.0)*
**Blood Pressure regulation**					
Hypertension	0.8 (0.6–1.2)	0.5 (0.3–0.8)*	0.7 (0.3–1.6)	0.9 (0.6–1.4)	0.9 (0.6–1.3)
Hypotension	1.1 (0.7–1.6)	1.4 (0.8–2.4)	1.0 (0.4–2.5)	0.9 (0.6–1.4)	1.1 (0.7–1.6)
Reverse Dipper >0% BP dip	1.3 (0.8–2.1)	1.6 (0.7–3.5)	1 (omitted)	1.3 (0.8–2.4)	1.3 (0.8–2.3)
Extreme Dipper > 20% BP dip	0.5 (0.3–0.8)*	0.4 (0.2–1.1)	1.1 (0.3–3.7)	0.4 (0.2–0.7)*	0.4 (0.2–0.8)*
Non-Dipper < 10% BP dip	1.6 (1.0–2.5)	2.3 (1.0–5.5)	0.6 (0.2–1.6)	2.0 (1.1–3.6)*	1.6 (1.0–2.8)
**Liver Health**					
Fibrosis risk: LSM ≥ 8	1.0 (0.7–1.5)	1.1 (0.6–2.1)	1.2 (0.4–3.6)	1.1 (0.7–1.8)	0.9 (0.6–1.5)
Fibrosis risk: LSM ≥ 12	1.0 (0.6–1.8)	1.2 (0.5–2.8)	Omitted	1.1 (0.6–2.1)	1.0 (0.6–1.9)
Fibrosis risk: Agile3 + ≥ 0.679	1.2 (0.7–1.9)	1.8 (0.8–3.8)	0.3 (0.0–2.7)	1.6 (0.9–2.6)	1.2 (0.7–2.1)
Cirrhosis risk: Agile4 ≥ 0.565	1.3 (0.8–2.2)	1.9 (0.8–4.0)	0.4 (0.1–3.2)	1.8 (1.0–3.2)*	1.3 (0.7–2.3)
Steatosis Risk, CAP ≥ 270	1.7 (1.2–2.5)*	1.8 (1.0–3.3)	1.2 (0.5–2.9)	1.6 (1.0–2.5)*	1.8 (1.1–2.7)*
**Sleep Apnea status**					
Any OSA, AHI ≥ 15 or CPAP treated	1.0 (0.7–1.5)	1.3 (0.7–2.6)	2.0 (0.7–5.6)	1.0 (0.6–1.6)	1.2 (0.7–1.9)
Severe OSA AHI ≥ 30	0.7 (0.4–1.3)	1.2 (0.5–3.2)	0.9 (0.2–4.3)	0.6 (0.3–1.3)	1.0 (0.5–2.0)

Odds of obesity-associated disease outcomes (binomial logistic regression) among individuals with different mental disorders compared to those without, adjusted for BMI, age, and sex. Significant associations (*p* < 0.05, chi-square test) are marked with an asterisk (*)

Abbreviations: *MD *Mental Disorder, *SCH *Schizophrenia, *BD *Bipolar Disorder, *DEP *Depression, *ANX *Anxiety, *IFG *Impaired Fasting Glucose, *HOMA2-IR *Homeostasis Model Assessment of Insulin Resistance, *MetS *Metabolic Syndrome, *LDL-C *Low-Density Lipoprotein Cholesterol, *HDL *High-Density Lipoprotein Cholesterol, *LSM *Liver Stiffness Measurement, *CAP *Controlled Attenuation Parameter, *AHI* Apnea-Hypopnea Index, *CPAP *Continuous Positive Airway Pressure

LDL-C did not differ between groups. However, OR of elevated triglycerides were significantly increased in AnyMD (OR = 2.0 [1.4–2.8]), SCH (OR = 2.1 [1.3–3.4]), DEP (OR = 2.4 [1.7–3.6]), and ANX (OR = 1.9 [1.3–2.9]). Reduced HDL-C levels were significantly more common in SCH (OR = 1.7 [1.0–2.8-0.8]). Elevated total cholesterol/HDL ratios were observed in AnyMD (OR = 1.9 [1.1–3.1]), SCH (OR = 2.38 [1.2–4.7]), and DEP (OR = 2.4 [1.4–4.2]). Lastly, the odds of elevated triglyceride-to-HDL ratios were significantly higher in AnyMD (OR = 2.6 [1.6–4.1]), SCH (OR = 3.45 [1.85–6.4]), DEP (OR = 3.1 [1.8–5.2]), and ANX (OR = 2.4 [1.4–4.0] (Table 3).

Only the SCH group showed decreased OR of hypertension (OR = 0.5 [0.28 − 0.82]), while there were no group differences on hypotension. Extreme nocturnal BP dipping was less frequent in the AnyMD (OR: 0.52 [0.3–0.9]), DEP (OR: 0.4 [0.2–0.8]) and ANX (OR: 0.4 [0.2–0.9]), and groups, whereas only the DEP group had higher OR of non-dipping (OR = 2.0 [1.1–3.6]) (Table 3).

The odds of cirrhosis, based on Agile-4, remained elevated in the DEP group in the adjusted analysis (OR: 1.8 [1.0–3.2]). Similarly, the odds of elevated steatosis risk remained higher in the AnyMD, DEP, and ANX groups (Table 3).

### The Contribution of BMI on Somatic Disease Outcomes

Higher BMI was significantly associated with an increased odds of all glucometabolic disease outcomes, with ORs ranging between 1.04 and 1.19 per BMI unit, except for prediabetes. Each unit increase in BMI was associated with a higher odds of MetS (OR = 1.06 [1.03–1.09]). No significant associations were found between BMI and blood lipid outcomes.

BMI was positively associated with hypertension (OR = 1.06 [1.04–1.09]) and negatively associated with hypotension (OR = 0.97 [0.94–0.99]). Nocturnal BP reverse dipping increased with higher BMI values (OR = 1.07 [1.02–1.11]), while neither BP non-dipping nor extreme dipping was significantly associated with BMI.

High LSM (≥ 8 and ≥12), high Agile3+ (≥ 0.679), and elevated steatosis risk (CAP ≥ 270) all increased with higher BMI, with ORs ranging between 1.03 and 1.14, while high Agile4 (≥ 0.565) was not significantly affected by BMI. OR for OSA (AHI ≥ 15 or CPAP treated) and severe OSA (AHI ≥ 30) increased with higher BMI values (OR = 1.08 [1.05–1.12] and 1.05 [1.01–1.09], respectively) (Supplementary Information: Table S3A- S3F and Fig. S2 for detail).

### Supplementary Analysis Stratified by Sex

In addition, as sex effects were frequently observed (Supplementary Tables S3A–S3F), an additional analysis stratified by sex was performed (Supplementary Table S4). Several outcomes showed notable sex-specific patterns, with significant associations observed mainly in women (e.g., high triglycerides, high triglyceride: HDL ratio, steatosis risk), while few reached significance in men.

### Psychotropic Drug Use and Obesity Associated Disease Outcomes

In the subsample of participants with MDs who were currently using psychotropic medication (*n* = 235), the pattern of associations with obesity-related somatic conditions closely resembled those observed in the main adjusted analysis. Notably, we observed increased odds for metabolic syndrome (OR: 1.7 [1.1–2.5]), elevated triglycerides (OR: 2.0 [1.4–3.0]), high total-to-HDL cholesterol ratio (OR: 2.3 [1.3–3.9]), elevated triglyceride-to-HDL ratio (OR: 3.0 [1.8–4.9]), and hepatic steatosis (CAP ≥ 270) (OR: 1.6 [1.1–2.4]).

Anxiolytics use increased the odds of diabetes (OR: 4.8 [1.3–18.1]), while antidepressants increased the odds of MetS (OR: 2.1 [1.2–3.5]).

Antidepressant use was positively associated with elevated triglycerides (OR: 2.6 [1.7–4.1]), elevated TotalC-HDL ratio (OR: 2.8 [1.5–5.1]), and elevated triglycerides-HDL ratio (OR: 3.2 [1.8–5.7]). Mood stabilizer were only significantly associated with higher OR of elevated triglycerides-HDL ratio (2.9 [1.4–5.9]). Antipsychotic use was linked to increased triglyceride levels (OR: 1.9 [1.2–3.0]), elevated TotalC-HDL ratio (OR: 2.2 [1.2–4.1]), and elevated triglycerides-HDL ratio (OR: 3.3 [1.9–5.9]), while hypnotics use was associated with elevated triglycerides-HDL ratio (OR: 4.7 [1.6–13.8]).

Mood stabilizers were negatively associated with hypertension (OR: 0.5 [0.3–0.9]). None of the psychotropic medications were associated with nocturnal BP dipping pattern (non-dipper, reverse dipper, extreme dipper). Antidepressant use significantly increased the OR for steatosis (CAP ≥ 270) (OR: 1.8 [1.1–3.0]). We found no significant associations between psychotropic drug use and sleep apnea outcomes (Supplementary Information: Table S5).

### Combined Model Adjusted for Psychotropics

After controlling for use of psychotropics, the association between having an MD (AnyMD) and elevated triglycerides (OR: 1.6 [1.1–2.4]), triglycerides-HDL-C ratio (OR: 2.3 [1.3–3.8]), liver steatosis risk (OR: 1.8 [1.1–2.8]) and extreme nocturnal BP dipping (OR: 0.5 [0.2–1.0.2.0]) remained significant, while Total-C-HDL ratio (OR: 1.3 [0.7–2.5]) and MetS (OR: 1.3 [0.8–2.0.8.0]) were no longer significantly associated with MD (Supplementary Information: Table S6).

## Discussion

We found that blood lipid abnormalities were particularly frequent among individuals with MDs. Additionally, participants with any MD had increased odds of liver steatosis. While an initial association was observed between MDs and metabolic syndrome (MetS), this association was no longer significant after adjusting for psychotropic medication use. Beyond blood lipid abnormalities, our findings suggest limited evidence of an elevated odds for obesity-associated diseases in individuals with MDs beyond what can be attributed to BMI.

### Psychotropic Medication

Participants with MDs, particularly those using antidepressants, had increased odds of MetS. However, this association disappeared after adjusting for antidepressant use, suggesting that psychotropic medication may either confound or mediate the relationship. While these drugs influence weight regulation and lipid metabolism, they may also mark a subgroup with more severe illness (Pillinger et al., 2020). In support of this, the subsample of 235 participants with current psychotropic use showed slightly stronger associations with several somatic conditions, such as MetS, elevated triglycerides, adverse lipid ratios, and hepatic steatosis, compared to the broader MD group. With the highest odds for users of antidepressants, potentially indicating greater clinical burden in those receiving pharmacological treatment. Notably, associations between MDs and elevated triglycerides, as well as adverse lipid ratios, remained significant even after adjusting for current psychotropic use, indicating that these relationships are not fully attributable to medication effects. However, it remains unclear whether the increased risk reflects pharmacological effects, greater illness severity, or both, particularly as carry-over effects from prior medication use cannot be ruled out.

In Denmark, the first-line treatment for anxiety, including single phobias, is cognitive behavioral therapy, with psychotropic medications, such as anxiolytics, rarely recommended or prescribed (Baandrup et al., 2021). Similarly, non-pharmacological interventions are the preferred approach for mild to moderate depression (Davidsen et al., 2019). Consequently, the relatively low rates of diagnosis-specific psychotropic prescriptions (e.g., anxiolytics for anxiety) observed in this study were expected.

### Blood Lipid Abnormalities and Their Influence on Health

Elevated triglycerides and an increased triglycerides-to-HDL ratio were among the most significantly altered lipid measures in individuals with MDs. Both have been linked to insulin resistance, increased cardiovascular risk, and all-cause mortality (Fulks et al., 2009; Kip et al., 2024; Kosmas et al., 2023). The triglycerides-to-HDL ratio, in particular, has emerged as a strong marker of metabolic dysfunction and an independent predictor of cardiovascular events (Kip et al., 2024; Kosmas et al., 2023).

With the exception of slightly increased odds of low HDL in participants with schizophrenia, neither low HDL nor high LDL was significantly more prevalent in individuals with MDs. LDL-C is widely used as a primary risk indicator for CVD (Bang, 2024; Mach et al., 2019); however, large-scale studies suggest a U-shaped relationship between LDL-C levels and both CVD and all-cause mortality, raising concerns about its use as a standalone predictive marker (Johannesen et al., 2020; Kip et al., 2024). This, in combination with present results, highlights the potential need to incorporate additional lipid metrics when assessing cardiovascular risk in individuals with MDs. Given the observed alterations in triglycerides and triglycerides-to-HDL ratios, and each association with cardiovascular outcomes (Kip et al., 2024; Kosmas et al., 2023; Toth et al., 2019) these may serve as important markers for lipid monitoring and metabolic health assessment in this population.

### BMI and the Link Between Mental Disorders and Obesity-Related Diseases

In this study, BMI emerged as a critical factor influencing the odds of obesity-associated diseases in individuals with and without MDs. Its impact spanned various conditions, including T2D, MetS, BP regulation, liver health, and sleep apnea. This underscores the importance of considering BMI in studies examining the relationship between MDs and physical health outcomes. Many studies on obesity-associated diseases do not account for BMI as a confounder, which may overestimate the independent effect of MDs on somatic health outcomes (Pérez-Piñar et al., 2016; Sharafkhaneh et al., 2005). When such studies are included in reviews and meta-analyses, this contribute to a potential misrepresentation of the relationship between MDs and obesity-related diseases (Dong et al., 2024; Macavei et al., 2016; Vancampfort et al., 2015). Neglecting BMI may lead to an overestimation of MD-related disease risk, conflating the effects of psychiatric conditions with those of obesity itself.

### Strength and Limitation

The present study exclusively included participants with obesity (BMI ≥ 30), ensuring that all individuals were at an elevated odds for obesity-associated diseases at baseline. While this strengthens the study’s relevance to a high-risk population, it also means that findings may not be generalizable to individuals with lower BMI. However, this design allows for greater precision in assessing the additional risk associated with MD diagnoses and psychotropic medications within a population already predisposed to obesity-related health conditions.

One major limitation of the present study is the reliance on self-reported data to categorize MDs. Based on our clinical observations patient can reliably self-identify as having schizophrenia or bipolar disorder whereas self-reported depression and anxiety could reflect emotional states rather than clinical diagnoses. One study found that self-reported depression had an accuracy of 74.2%, with 25.8% false positives and 18.9% false negatives, suggesting that self-reports are fairly reliable but not without misclassification (Turon et al., 2019). The high rates of unemployment and psychotropic medication use across MD groups, along with the ability of study nurses to validate self-reported diagnoses, suggest that diagnosis misclassification may not have been a significant issue.

Another important limitation is the absence of detailed lifestyle and socioeconomic data, such as smoking status, physical activity, and income or education level. These factors are known to influence cardiometabolic health (Kim et al., 2018). While we adjusted for BMI, age, and sex in all models, residual confounding from unmeasured variables remains a possibility and may have attenuated or inflated observed associations.

This study included numerous analyses to evaluate the relationship between MDs, psychotropic medications, and obesity-associated diseases. To reduce the likelihood of type II errors we chose not to adjust for multiplicity in the primary analyses. Post hoc adjustments (Supplementary Table S7) showed only blood lipid abnormalities and liver steatosis remained significantly associated with MD.

The cross-sectional design precludes establishing causal relationships between MDs, psychotropic medications, and obesity-associated diseases. To address potential selection bias, we also compared baseline characteristics and disease prevalence of excluded participants with those of included participants (Supplementary Tables S8A–S8B). The excluded group generally resembled the NoMD group, with values often falling between NoMD and AnyMD, and no consistent pattern suggesting systematic bias.

## Conclusion

Aside from blood lipid abnormalities and elevated risk of steatosis, we found little evidence of increased risk for obesity-related diseases in individuals with MDs beyond the elevated risk attributable to obesity itself. Our results underscore the importance of considering BMI when evaluating obesity associated disease prevalence in people with MDs. Addressing obesity may the most effective strategy for reducing health risks in this population, while specific attention on managing blood lipid abnormalities may be required.

## Supplementary Information

Below is the link to the electronic supplementary material.

Supplementary Material 1 (DOCX 442 KB)

## Data Availability

Data are available from the corresponding author on reasonable request.
