# Supplementary material for: Obesity Associated Disease in People with Mental Disorders: the Role of Psychotropic Medication and BMI
Source: Community Ment Health J. 2025 Nov 11;62(3):432–42. doi: 10.1007/s10597-025-01536-y (PMC12963104; doi:10.1007/s10597-025-01536-y)
Supplement: Supplementary file 1 — (DOCX 442 KB) [file 10597_2025_1536_MOESM1_ESM.docx]

# **Supporting information** - Obesity Associated Disease in People with Mental Disorders: The Impact of Psychotropic Medication and BMI

Corresponding Author:

Mikkel EI Kolind

E-Mail: [Mikkel.Emil.Iwanoff.Kolind@rsyd.dk](mailto:Mikkel.Emil.Iwanoff.Kolind@rsyd.dk)

Haraldsgade 7, DK-6700,

Esbjerg Department of Endocrinology,

Hospital of South West Jutland, Esbjerg, Denmark

**Description:** This document contains tables, figures, and additional statistics not included in the main paper (Obesity Associated Disease in People with Mental Disorders: The Impact of Psychotropic Medication and BMI).

- **Tables S1A-1** provide detailed information on the types and quantities of psychotropic medications used by participants in the South Danish Initiative cohort.
- **Figure S1A-2** UpSet plot illustrating patterns of **polypharmacy** among participants with mental disorders.
- **Tables S1B-S1E** provide detailed information on non-psychotropic types and quantities of medications used by participants in the South Danish Initiative cohort.
- **Table S2** displays results from fasted blood samples, used to categorize disease risks related to glucometabolic health, blood lipid abnormalities, and liver disease.
- **Tables S3A–S3F** include additional statistics supporting the models used to generate Table 3 in the main paper. **Figures 1A–1F** visually represent the effects of BMI in these models.
- **Table S4** details odds of somatic disease stratified by sex adjusted for age and BMI
- **Table S5** and **Table S6** outline models evaluating:
  - The effect of psychotropic medications on somatic disease risk (Table S4).
  - The combined effect of psychotropic medications and mental disorders on somatic disease risk (Table S5).
- **Table S7** contains post hoc test results adjusting for multiplicity using the Benjamini-Hochberg method.
- **Table S8A and S8B** details characteristics of participants excluded from analyses

# Medication details and user count

**Table S1A-1.** Psychotropic medication use in participants with mental disorders

| **Medication group** | **Medication Type** | **AnyMD (n=)** | **SCH (n=)** | **BD (n=)** | **DEP (n=)** | **ANX (n=)** | **Medications Included (ATC Code and Name)** | **Treatment Indication** |
| --- | --- | --- | --- | --- | --- | --- | --- | --- |
| Antidepressant | Selective Serotonin Reuptake Inhibitors (SSRIs) | 77 | 26 | 6 | 60 | 58 | Citalopram (N06AB04), Escitalopram (N06AB10), Fluoxetine (N06AB03), Sertraline (N06AB06) | Depression, anxiety disorders, OCD, PTSD |
| Antidepressant | Serotonin and Noradrenaline Reuptake Inhibitors (SNRIs) | 60 | 12 | 6 | 51 | 40 | Duloxetine (N06AX21), Venlafaxine (N06AX16), Vortioxetine (N06AX26) | Major depressive disorder, anxiety disorders, chronic pain |
| Antidepressant | Tricyclic Antidepressants (TCAs) | 4 | 1 | 1 | 2 | 3 | Amitriptyline (N06AA09), Nortriptyline (N06AA10), Chlorprothixen (N05AF03) | Depression, chronic pain, migraine prevention |
| Antidepressant | Monoamine Oxidase Inhibitors (MAOIs) | 1 | 0 | 0 | 1 | 1 | Isocarboxazid (N06AF01) | Treatment-resistant and atypical depression |
| Antidepressant | Norepinephrine Reuptake Inhibitors (Norepi-RI) | 2 | 0 | 0 | 1 | 2 | Atomoxetine (N06BA09) | Primarily ADHD |
| Antidepressant | Atypical Antidepressants | 11 | 0 | 1 | 9 | 8 | Mirtazapine (N06AX11), Bupropion (N06AX12), Agomelatine (N06AX22) | Depression, smoking cessation |
| Antipsychotics | First-Generation (Typical) Antipsychotics | 13 | 10 | 0 | 4 | 11 | Haloperidol (N05AD01), Perphenazine (N05AB03), Zuclopenthixol (N05AF05) | Schizophrenia, acute psychosis, bipolar disorder |
| Antipsychotics | Second-Generation (Atypical) Antipsychotics | 128 | 75 | 17 | 70 | 97 | Aripiprazole (N05AX12), Clozapine (N05AH02), Olanzapine (N05AH03), Quetiapine (N05AH04), Risperidone (N05AX08), Paliperidone (N05AX13), Ziprasidone (N05AE04), Sertindol (N05AE03) | Schizophrenia, bipolar disorder, severe depression (adjunct) |
| Anxiolytics | Benzodiazepine Anxiolytics | 10 | 3 | 1 | 5 | 9 | Alprazolam (N05BA12), Diazepam (N05BA01), Oxazepam (N05BA04) | Short-term anxiety, panic disorders, insomnia |
| Anxiolytics | Non-Benzodiazepine Anxiolytics | 1 | 0 | 0 | 1 | 1 | Buspirone (N05BE01) | Generalized anxiety disorder |
| Mood stabilizers | Lithium | 7 | 1 | 6 | 3 | 3 | Lithium (N05AN01) | Bipolar disorder, augmentation in depression |
| Mood stabilizers | Anticonvulsants used as Mood Stabilizers | 65 | 25 | 14 | 47 | 46 | Lamotrigine (N03AX09), Valproic Acid (N03AG01), Gabapentin (N03AX12), Pregabalin (N03AX16), Topiramate (N03AX11) | Bipolar disorder, seizure disorders, neuropathic pain |
| Hypnotics | Z-Drugs (Non-Benzodiazepine Sedatives) | 10 | 4 | 1 | 9 | 8 | Zolpidem (N05CF02), Zopiclone (N05CF01) | Short-term insomnia |
| Hypnotics | Benzodiazepine Sedatives | 9 | 3 | 1 | 4 | 8 | Midazolam (N05CD08), Flurazepam (N05CD01) | Insomnia, preoperative sedation |
| Stimulants | Stimulants | 10 | 3 | 1 | 4 | 9 | Methylphenidate (N06BA04) | ADHD, treatment-resistant depression |
| Other | Antihistamines (Psychiatric Use) | 4 | 2 | 0 | 3 | 4 | Hydroxyzine (N05BB01) | Anxiety, sedative use |

The table presents the distribution of psychotropic medication use across different mental disorder (MD) groups, including schizophrenia (SCH), bipolar disorder (BD), depression (DEP), and anxiety disorders (ANX). The listed medications are categorized by their pharmacological class, along with their corresponding Anatomical Therapeutic Chemical (ATC) classification codes and primary treatment indications.


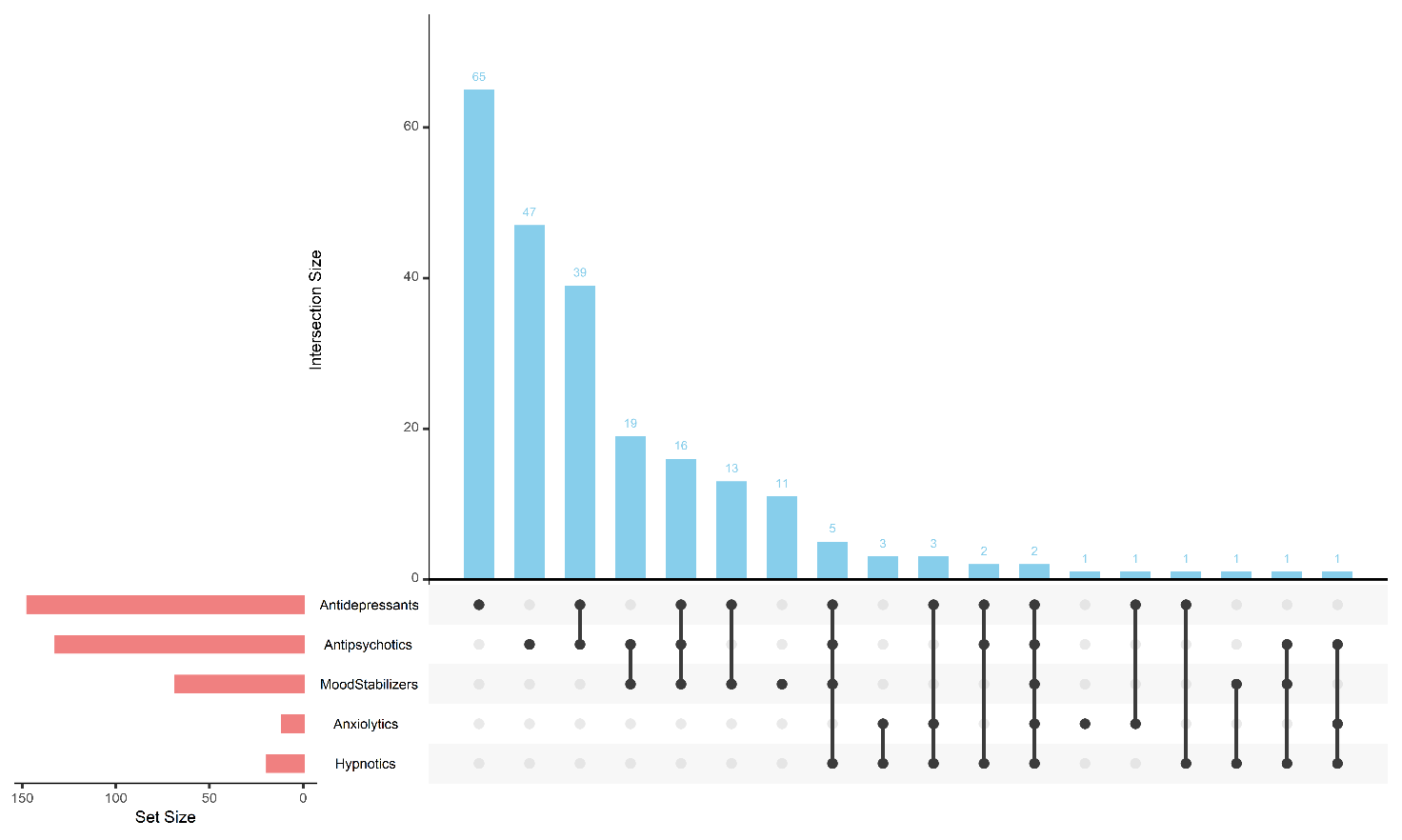


**Figure S1A-2.** *UpSet plot of psychotropic medication usage among participants with mental disorders.* The plot displays the combinations and frequencies of psychotropic medications prescribed to participants. The horizontal bars on the left represent the total number of participants prescribed each medication type individually, while the vertical bars indicate the size of each combination of medications used, with intersecting lines connecting the corresponding medication categories.

**Table S1B.** Lipid medication use in participants with and without mental disorders

| **Medication Group** | **NoMD (n=)** | **AnyMD (n=)** | **SCH (n=)** | **BD (n=)** | **DEP (n=)** | **ANX (n=)** | **Medications Included (ATC Code and Name)** | **Treatment Indication** |
| --- | --- | --- | --- | --- | --- | --- | --- | --- |
| **Statins** | 46 | 59 | 19 | 6 | 37 | 42 | Simvastatin (C10AA01), Atorvastatin (C10AA05), Rosuvastatin (C10AA07) | High cholesterol, cardiovascular risk reduction |
| **Fibrates** | 0 | 1 | 0 | 0 | 1 | 1 | Fenofibrate (C10AB05), Gemfibrozil (C10AB02) | Hypertriglyceridemia, mixed dyslipidemia |
| **PCSK9 Inhibitors** | 1 | 0 | 0 | 0 | 0 | 0 | Alirocumab (C10AX13), Evolocumab (C10AX14) | High cholesterol, cardiovascular risk reduction in statin-intolerant patients |
| **Ezetimibe** | 0 | 0 | 0 | 0 | 0 | 0 | Ezetimibe (C10AX09) | High cholesterol, often used in combination with statins |
| **Beta-Blockers** | 17 | 24 | 6 | 2 | 17 | 17 | Metoprolol (C07AB02), Propranolol (C07AA05), Bisoprolol (C07AB07) | Hypertension, angina, heart failure, anxiety symptoms |

Use of lipid lowering medication in participants with and without mental disorders across different mental disorder (MD) groups, including schizophrenia (SCH), bipolar disorder (BD), depression (DEP), and anxiety disorders (ANX). The listed medications are categorized by their pharmacological class, along with their corresponding Anatomical Therapeutic Chemical (ATC) classification codes and primary treatment indications.

**Table S1D**. Diabetes medication use in participants with and without mental disorders

| **Medication Group** | **NoMD (n=)** | **AnyMD (n=)** | **SCH (n=)** | **BD (n=)** | **DEP (n=)** | **ANX (n=)** | **Medications Included (ATC Code and Name)** | **Treatment Indication** |
| --- | --- | --- | --- | --- | --- | --- | --- | --- |
| **Insulin Analogues** | 4 | 1 | 0 | 0 | 1 | 1 | Insulin aspart (A10AB01), Insulin lispro (A10AB04), Insulin glargine (A10AE04), Insulin degludec (A10AE06) | Diabetes mellitus (primarily Type 1 and 2) |
| **Metformin** | 23 | 32 | 9 | 1 | 20 | 22 | Metformin (A10BA02) | Type 2 diabetes |
| **SGLT-2 Inhibitors** | 8 | 4 | 2 | 0 | 2 | 3 | Dapagliflozin (A10BK01), Empagliflozin (A10BK03) | Type 2 diabetes, weight management |
| **GLP-1 Receptor Agonists** | 26 | 29 | 10 | 1 | 15 | 26 | Liraglutide (A10BJ02), Semaglutide (A10BJ06), Exenatide (A10BJ01) | Type 2 diabetes, weight management |
| **DPP-4 Inhibitors** | 1 | 0 | 0 | 0 | 0 | 0 | Sitagliptin (A10BH01), Linagliptin (A10BH05), Saxagliptin (A10BH03) | Type 2 diabetes |
| **Sulfo-nylureas** | 0 | 0 | 0 | 0 | 0 | 0 | Glibenclamide (A10BB01), Glipizide (A10BB07), Gliclazide (A10BB09) | Type 2 diabetes |
| **Thiazo-lidinediones** | 0 | 0 | 0 | 0 | 0 | 0 | Pioglitazone (A10BG03), Rosiglitazone (A10BG02) | Type 2 diabetes |

Use of Diabetes medication in participants with and without mental disorders across different mental disorder (MD) groups, including schizophrenia (SCH), bipolar disorder (BD), depression (DEP), and anxiety disorders (ANX). The listed medications are categorized by their pharmacological class, along with their corresponding Anatomical Therapeutic Chemical (ATC) classification codes and primary treatment indications.

**Table S1E**. Blood Pressure medication use in participants with and without mental disorders

| **Medication Group** | **NoMD (n=)** | **AnyMD (n=)** | **SCH (n=)** | **BD (n=)** | **DEP (n=)** | **ANX (n=)** | **Medications Included (ATC Code and Name)** | **Treatment Indication** |
| --- | --- | --- | --- | --- | --- | --- | --- | --- |
| **Anti-hypertensiva** | 84 | 82 | 17 | 7 | 56 | 55 | Amlodipine (C08CA01), Enalapril (C09AA02), Losartan (C09CA01), Ramipril (C09AA05), Hydrochlorothiazide (C03AA03), Furosemide (C03CA01) | General antihypertensive use |
| **Diuretics with BP effect** | 27 | 22 | 7 | 2 | 15 | 14 | Hydrochlorothiazide (C03AA03), Furosemide (C03CA01), Spironolactone (C03DA01), Indapamide (C03BA11) | Hypertension, edema |
| **ACE Inhibitors** | 32 | 28 | 3 | 4 | 16 | 20 | Enalapril (C09AA02), Ramipril (C09AA05), Captopril (C09AA01) | Hypertension, heart failure |
| **Calcium Channel Blockers** | 18 | 25 | 3 | 2 | 22 | 18 | Amlodipine (C08CA01), Nifedipine (C08CA05), Verapamil (C08DA01), Diltiazem (C08DB01) | Hypertension, angina |
| **Beta-Blockers** | 17 | 24 | 6 | 2 | 17 | 17 | Metoprolol (C07AB02), Atenolol (C07AB03), Propranolol (C07AA05), Bisoprolol (C07AB07) | Hypertension, arrhythmia |

Use of Blood pressure medication in participants with and without mental disorders across different mental disorder (MD) groups, including schizophrenia (SCH), bipolar disorder (BD), depression (DEP), and anxiety disorders (ANX). The listed medications are categorized by their pharmacological class, along with their corresponding Anatomical Therapeutic Chemical (ATC) classification codes and primary treatment indications.

# Blood Tests

**Table S2** Fasted blood samples with comparison

| Outcome Variable | No MD (n=314)  median (q1,q3) | Any MD (n=345)  median (q1,q3) | SCH (n=97)  median (q1,q3) | BD (n=27)  median (q1,q3) | DEP (n=204)  median (q1,q3) | ANX (n=246)  median (q1,q3) |
| --- | --- | --- | --- | --- | --- | --- |
| glucose (mmol/L) | 5.80 (5.40, 6.40) | 5.80 (5.40, 6.30) (p=0.803) | 5.80 (5.40, 6.30) (p=0.962) | 5.70 (5.20, 6.10) (p=0.362) | 5.80 (5.40, 6.30) (p=0.907) | 5.80 (5.40, 6.30) (p=0.456) |
| hba1c (mmol/mol) | 36.50 (34.00, 40.00) | 37.00 (34.00, 40.00) (p=0.312) | 37.00 (34.00, 40.00) (p=0.606) | 35.00 (33.00, 38.00) (p=0.223) | 37.00 (35.00, 40.00) (p=0.230) | 37.00 (34.00, 40.00) (p=0.657) |
| insulin (pmol/L) | 145.00 (102.00, 215.00) | **171.00 (117.00, 249.00) (p=0.001)** | **176.50 (134.00, 253.00) (p=0.003)** | 141.00 (88.00, 218.00) (p=0.844) | **166.00 (117.00, 248.00) (p=0.004)** | **173.00 (119.00, 249.00) (p=0.001)** |
| Cholesterol (mmol/L) | 4.70 (4.00, 5.30) | 4.60 (4.10, 5.30) (p=0.684) | 4.70 (4.20, 5.40) (p=0.193) | 4.60 (4.30, 5.30) (p=0.319) | 4.60 (4.15, 5.25) (p=0.719) | 4.60 (4.10, 5.30) (p=0.800) |
| LDL (mmol/L) | 3.20 (2.60, 3.80) | 3.30 (2.70, 3.90) (p=0.670) | 3.35 (2.80, 4.10) (p=0.114) | 3.20 (2.70, 4.10) (p=0.473) | 3.20 (2.70, 3.90) (p=0.705) | 3.30 (2.60, 3.80) (p=0.943) |
| HDL (mmol/L) | 1.20 (1.00, 1.40) | **1.10 (0.90, 1.30) (p=0.003)** | **1.00 (0.90, 1.20) (<0.001)** | 1.20 (1.00, 1.40) (p=0.438) | **1.10 (0.90, 1.30) (p=0.004)** | **1.10 (0.90, 1.30) (<0.001)** |
| Triglycerides (mmol/L) | 1.30 (1.00, 1.77) | **1.50 (1.07, 2.20) (<0.001)** | **1.53 (1.08, 2.39) (p=0.002)** | 1.46 (1.00, 2.25) (p=0.622) | **1.60 (1.14, 2.25) (<0.001)** | **1.49 (1.08, 2.16) (p=0.003)** |
| Platelets (x10^9/l) | 266.00 (232.00, 302.00) | **282.00 (240.00, 328.00) (<0.001)** | 269.50 (233.00, 319.00) (p=0.297) | **306.00 (260.00, 340.00) (p=0.003)** | **278.00 (236.00, 326.00) (p=0.009)** | **281.00 (239.00, 324.00) (p=0.002)** |
| ASAT (IU/L) | 22.50 (16.00, 27.00) | 23.00 (15.00, 27.00) (p=0.773) | 23.00 (15.00, 27.00) (p=0.930) | 22.50 (15.00, 26.00) (p=0.478) | 23.00 (15.00, 27.00) (p=0.944) | 23.00 (15.50, 27.00) (p=0.693) |
| ALAT (IU/L) | 27.00 (20.00, 42.00) | 27.00 (20.00, 42.00) (p=0.574) | 27.50 (21.00, 45.00) (p=0.186) | 22.00 (17.00, 32.00) (p=0.079) | 28.50 (21.00, 44.00) (p=0.253) | 27.00 (20.00, 40.00) (p=0.667) |

Results from fasted blood samples used to calculate glucometabolic and liver health outcomes. All comparisons are performed with the NoMD group as reference. Values are presented as median (lower, upper quartile). Bold text indicates a statistical significant difference based on mann whitney U statistics (p<0.05). Abbreviations: MD = Mental Disorder, NoMD = No Mental Disorder, AnyMD = Any Mental Disorder, SCH = Schizophrenia, BD = Bipolar Disorder, DEP = Depression, ANX = Anxiety Disorders, LDL = Low-Density Lipoprotein, HDL = High-Density Lipoprotein, ASAT = Aspartate Aminotransferase, ALAT = Alanine Aminotransferase.

# Expanded model details and analyses

**Table S3A** Model details Glucometabolic Health

|  | **Any MD**  **OR (95% CI)** | **SCH**  **OR (95% CI)** | **BD**  **OR (95% CI)** | **DEP**  **OR (CI95%)** | **ANX**  **OR (95% CI)** |
| --- | --- | --- | --- | --- | --- |
| IFG | 1.22 (0.83–1.79) | 1.93 (1.09–3.44)* | 1.02 (0.38–2.78) | 1.30 (0.84–2.02) | 1.07 (0.69–1.66) |
| - BMI | 1.04 (1.02–1.07)*** | 1.07 (1.03–1.10)*** | 1.07 (1.03–1.11)** | 1.05 (1.02–1.08)** | 1.06 (1.03–1.09)*** |
| - Age | 1.04 (1.02–1.06)*** | 1.06 (1.03–1.08)*** | 1.05 (1.03–1.08)*** | 1.04 (1.02–1.06)*** | 1.05 (1.03–1.07)*** |
| - Sex | 0.42 (0.28–0.63)*** | 0.46 (0.29–0.75)** | 0.38 (0.22–0.66)*** | 0.41 (0.27–0.64)*** | 0.44 (0.28–0.67)*** |
| Prediabetes | 0.71 (0.28–1.78) | 1.69 (0.52–5.50) | 1.00 (omitted) | 0.61 (0.20–1.85) | 0.61 (0.20–1.89) |
| - BMI | 1.04 (0.98–1.10) | 1.07 (1.00–1.14)* | 1.10 (1.01–1.20)* | 1.05 (0.98–1.12) | 1.05 (0.98–1.12) |
| - Age | 1.04 (0.99–1.08) | 1.05 (0.99–1.10) | 1.06 (0.98–1.13) | 1.05 (1.00–1.11)* | 1.06 (1.01–1.12)* |
| - Sex | 1.83 (0.60–5.60) | 2.62 (0.73–9.44) | 3.29 (0.68–15.81) | 2.27 (0.63–8.22) | 2.33 (0.64–8.45) |
| Diabetes | 1.23 (0.73–2.06) | 1.39 (0.64–3.05) | 0.73 (0.16–3.35) | 1.08 (0.60–1.96) | 1.50 (0.85–2.65) |
| - BMI | 1.05 (1.02–1.08)** | 1.05 (1.01–1.10)* | 1.07 (1.01–1.12)* | 1.05 (1.01–1.09)** | 1.04 (1.01–1.08)* |
| - Age | 1.05 (1.03–1.08)*** | 1.05 (1.02–1.09)*** | 1.06 (1.02–1.10)** | 1.05 (1.02–1.08)*** | 1.06 (1.03–1.09)*** |
| - Sex | 0.70 (0.41–1.18) | 0.74 (0.39–1.41) | 0.77 (0.37–1.60) | 0.74 (0.41–1.34) | 0.63 (0.36–1.11) |
| HOMA2-IR > 1.4 | 1.12 (0.61–2.06) | 1.15 (0.42–3.13) | 1.08 (0.29–4.07) | 1.49 (0.68–3.27) | 1.28 (0.63–2.58) |
| - BMI | 1.19 (1.12–1.28)*** | 1.17 (1.08–1.27)*** | 1.17 (1.08–1.28)*** | 1.17 (1.09–1.26)*** | 1.20 (1.11–1.29)*** |
| - Age | 0.98 (0.96–1.01) | 0.97 (0.94–1.01) | 0.97 (0.93–1.01) | 0.98 (0.95–1.01) | 0.99 (0.96–1.02) |
| - Sex | 0.13 (0.04–0.42)** | 0.05 (0.01–0.38)** | 0.06 (0.01–0.45)** | 0.16 (0.05–0.55)** | 0.05 (0.01–0.35)*** |

Odds of Glucometabolic outcomes (binomial logistic regression) among participants with and without mental disorders, adjusted for BMI, age, and sex. Significant associations (p < 0.05,0.01,0.001) are marked with an asterisk (*,**,***). Abbreviations: IFG = Impaired Fasting Glucose; HOMA2-IR = Homeostasis Model Assessment of Insulin Resistance.

**Table S3B** Model details Metabolic Syndrome (MetS)

|  | **Any MD**  **OR (95% CI)** | **SCH**  **OR (95% CI)** | **BD**  **OR (95% CI)** | **DEP**  **OR (CI95%)** | **ANX**  **OR (95% CI)** |
| --- | --- | --- | --- | --- | --- |
| **Mets** | 1.55 (1.07–2.25)* | 1.69 (0.95–2.99) | 0.86 (0.36–2.05) | 1.78 (1.14–2.77)** | 1.48 (0.97–2.26) |
| - BMI | 1.06 (1.03–1.09)*** | 1.07 (1.03–1.11)*** | 1.08 (1.04–1.13)*** | 1.05 (1.02–1.09)** | 1.08 (1.04–1.11)*** |
| - Age | 1.04 (1.02–1.05)*** | 1.04 (1.02–1.06)*** | 1.06 (1.03–1.08)*** | 1.04 (1.02–1.06)*** | 1.04 (1.02–1.06)*** |
| - Sex | 0.44 (0.28–0.69)*** | 0.41 (0.24–0.70)** | 0.38 (0.20–0.70)** | 0.44 (0.27–0.73)** | 0.41 (0.25–0.67)*** |

Odds of Metabolic Syndrome (binomial logistic regression) among participants with and without mental disorders, adjusted for BMI, age, and sex. Significant associations (p < 0.05,0.01,0.001) are marked with an asterisk (*,**,***). Abbreviations: MetS = Metabolic Syndrome.

**Table S3C** Model details Blood Lipid Abnormalities

|  | **Any MD**  **OR (95% CI)** | **SCH**  **OR (95% CI)** | **BD**  **OR (95% CI)** | **DEP**  **OR (CI95%)** | **ANX**  **OR (95% CI)** |
| --- | --- | --- | --- | --- | --- |
| **LDL-C ≥ 3 mmol/l** | 0.91 (0.65–1.27) | 1.36 (0.81–2.28) | 0.63 (0.28–1.45) | 0.96 (0.65–1.41) | 0.89 (0.61–1.29) |
| - BMI | 1.01 (0.99–1.03) | 1.02 (0.99–1.05) | 1.04 (1.01–1.08)* | 1.01 (0.98–1.03) | 1.01 (0.99–1.04) |
| - Age | 1.00 (0.99–1.02) | 1.01 (0.99–1.03) | 1.02 (1.00–1.05)* | 1.01 (1.00–1.03) | 1.00 (0.99–1.02) |
| - Sex | 1.28 (0.90–1.83) | 1.59 (1.03–2.45)* | 2.03 (1.23–3.33)** | 1.41 (0.95–2.10) | 1.51 (1.03–2.21)* |
| **Triglycerides ≥ 1.7 mmol/l** | 1.99 (1.42–2.80)*** | 2.08 (1.26–3.43)** | 1.02 (0.41–2.55) | 2.44 (1.65–3.61)*** | 1.94 (1.33–2.85)** |
| - BMI | 1.00 (0.98–1.02) | 0.99 (0.96–1.02) | 1.00 (0.96–1.04) | 0.99 (0.97–1.02) | 1.00 (0.98–1.03) |
| - Age | 1.02 (1.00–1.03)* | 1.01 (0.99–1.03) | 1.03 (1.00–1.05)* | 1.02 (1.01–1.04)** | 1.02 (1.00–1.03)* |
| - Sex | 0.48 (0.33–0.68)*** | 0.48 (0.31–0.74)** | 0.44 (0.27–0.73)** | 0.45 (0.30–0.68)*** | 0.44 (0.30–0.65)*** |
| **HDL-C < 1.3 (M) or <1.0 (FM)** | 1.16 (0.83–1.63) | 1.68 (1.00–2.82)* | 0.88 (0.39–1.99) | 1.39 (0.94–2.06) | 1.30 (0.89–1.88) |
| - BMI | 1.02 (1.00–1.04) | 1.03 (1.00–1.07)* | 1.04 (1.00–1.07)* | 1.01 (0.98–1.04) | 1.02 (0.99–1.04) |
| - Age | 0.96 (0.94–0.97)*** | 0.96 (0.94–0.98)*** | 0.97 (0.95–0.99)** | 0.96 (0.94–0.98)*** | 0.96 (0.95–0.98)*** |
| - Sex | 2.07 (1.43–2.98)*** | 1.87 (1.21–2.90)** | 1.86 (1.14–3.05)* | 2.12 (1.41–3.17)*** | 1.84 (1.24–2.72)** |
| **Total-C/HDL Ratio > 6.0** | 1.87 (1.12–3.12)* | 2.38 (1.20–4.72)* | 1.07 (0.23–4.94) | 2.43 (1.40–4.23)** | 1.75 (0.99–3.07) |
| - BMI | 0.99 (0.96–1.02) | 1.01 (0.97–1.06) | 1.01 (0.96–1.07) | 0.98 (0.95–1.02) | 1.00 (0.96–1.04) |
| - Age | 0.99 (0.97–1.01) | 0.99 (0.97–1.02) | 1.01 (0.98–1.05) | 0.98 (0.96–1.01) | 0.98 (0.96–1.01) |
| - Sex | 0.33 (0.20–0.55)*** | 0.32 (0.17–0.59)*** | 0.44 (0.20–0.95)* | 0.38 (0.22–0.66)** | 0.36 (0.21–0.63)*** |
| **HDL-Triglycerides ratio ≥ 2.97 (M) or ≥ 2.23 (FM)** | 2.55 (1.59–4.08)*** | 3.45 (1.85–6.44)*** | 1.90 (0.59–6.14) | 3.06 (1.82–5.15)*** | 2.37 (1.41–3.98)** |
| - BMI | 1.00 (0.97–1.03) | 1.00 (0.96–1.04) | 1.02 (0.97–1.08) | 1.00 (0.97–1.03) | 1.00 (0.97–1.03) |
| - Age | 0.99 (0.97–1.01) | 1.00 (0.97–1.02) | 1.01 (0.98–1.04) | 1.00 (0.98–1.02) | 0.98 (0.96–1.01) |
| - Sex | 0.49 (0.31–0.77)** | 0.50 (0.28–0.90)* | 0.48 (0.23–0.99)* | 0.49 (0.29–0.83)** | 0.45 (0.27–0.75)** |

Odds of Blood Lipid Abnormalities (binomial logistic regression) among participants with and without mental disorders, adjusted for BMI, age, and sex. Significant associations (p < 0.05,0.01,0.001) are marked with an asterisk (*,**,***). Abbreviations: LDL-C = Low-Density Lipoprotein Cholesterol; HDL = High-Density Lipoprotein Cholesterol.

**Table S3D** Model details Blood Pressure Outcomes

|  | **Any MD**  **OR (95% CI)** | **SCH**  **OR (95% CI)** | **BD**  **OR (95% CI)** | **DEP**  **OR (CI95%)** | **ANX**  **OR (95% CI)** |
| --- | --- | --- | --- | --- | --- |
| Hypertension | 0.84 (0.59–1.19) | 0.48 (0.28–0.82)** | 0.68 (0.29–1.61) | 0.93 (0.62–1.39) | 0.87 (0.59–1.28) |
| - BMI | 1.06 (1.04–1.09)*** | 1.07 (1.03–1.10)*** | 1.09 (1.05–1.13)*** | 1.07 (1.04–1.10)*** | 1.07 (1.04–1.10)*** |
| - Age | 1.06 (1.05–1.08)*** | 1.06 (1.04–1.09)*** | 1.07 (1.05–1.10)*** | 1.07 (1.05–1.09)*** | 1.07 (1.05–1.08)*** |
| - Sex | 0.37 (0.25–0.55)*** | 0.32 (0.20–0.53)*** | 0.32 (0.18–0.56)*** | 0.33 (0.21–0.52)*** | 0.36 (0.23–0.56)*** |
| Hypotension | 0.98 (0.68–1.42) | 1.18 (0.70–2.00) | 1.03 (0.42–2.53) | 0.82 (0.53–1.27) | 0.98 (0.65–1.47) |
| - BMI | 0.97 (0.94–0.99)** | 0.98 (0.94–1.01) | 0.95 (0.92–0.99)* | 0.96 (0.93–0.99)* | 0.97 (0.94–0.99)* |
| - Age | 0.97 (0.95–0.98)*** | 0.96 (0.94–0.98)*** | 0.95 (0.93–0.97)*** | 0.96 (0.94–0.98)*** | 0.96 (0.95–0.98)*** |
| - Sex | 2.88 (1.79–4.62)*** | 2.56 (1.50–4.37)*** | 2.17 (1.17–4.03)** | 2.83 (1.66–4.82)*** | 2.48 (1.51–4.06)*** |
| Reverse Dipper >0% BP dip | 1.12 (0.60–2.10) | 2.40 (0.98–5.88) | 1.00 (omitted) | 1.17 (0.58–2.36) | 1.11 (0.55–2.22) |
| - BMI | 1.07 (1.02–1.11)** | 1.06 (1.00–1.12) | 1.08 (1.00–1.16)* | 1.07 (1.02–1.12)** | 1.05 (1.01–1.10)* |
| - Age | 1.01 (0.99–1.04) | 1.02 (0.98–1.05) | 0.99 (0.96–1.04) | 1.02 (0.98–1.05) | 1.01 (0.98–1.04) |
| - Sex | 1.02 (0.52–2.00) | 1.03 (0.47–2.30) | 0.66 (0.27–1.63) | 0.95 (0.46–1.97) | 1.05 (0.51–2.16) |
| Extreme Dipper >20% BP dip | 0.52 (0.29–0.93)* | 0.41 (0.15–1.17) | 1.56 (0.42–5.74) | 0.36 (0.17–0.78)** | 0.44 (0.22–0.87)* |
| - BMI | 0.97 (0.92–1.01) | 0.99 (0.93–1.05) | 0.99 (0.93–1.05) | 0.97 (0.92–1.02) | 0.97 (0.93–1.02) |
| - Age | 0.97 (0.94–0.99)** | 0.97 (0.94–1.00)* | 0.97 (0.94–1.00)* | 0.97 (0.94–1.00)* | 0.96 (0.94–0.99)** |
| - Sex | 1.07 (0.57–2.01) | 1.37 (0.66–2.88) | 1.32 (0.60–2.89) | 1.26 (0.62–2.56) | 0.88 (0.46–1.70) |
| Non-Dipper <10% BP dip | 1.59 (0.99–2.54) | 2.34 (1.00–5.50) | 0.38 (0.11–1.28) | 2.01 (1.14–3.56)* | 1.65 (0.97–2.81) |
| - BMI | 1.01 (0.98–1.04) | 1.01 (0.96–1.06) | 1.00 (0.95–1.05) | 1.01 (0.97–1.05) | 1.01 (0.97–1.05) |
| - Age | 1.00 (0.98–1.03) | 1.00 (0.98–1.03) | 1.01 (0.98–1.03) | 1.00 (0.98–1.02) | 1.01 (0.99–1.03) |
| - Sex | 1.29 (0.78–2.11) | 1.14 (0.62–2.08) | 1.36 (0.71–2.58) | 1.43 (0.83–2.47) | 1.55 (0.91–2.62) |

Odds of Blood Pressure Outcomes (binomial logistic regression) among participants with and without mental disorders, adjusted for BMI, age, and sex. Significant associations (p < 0.05,0.01,0.001) are marked with an asterisk (*,**,***). Abbreviations: BP = Blood pressure.

**Supplementary Table S3E** Model details Liver Health Outcomes

|  | **Any MD**  **OR (95% CI)** | **SCH**  **OR (95% CI)** | **BD**  **OR (95% CI)** | **DEP**  **OR (CI95%)** | **ANX**  **OR (95% CI)** |
| --- | --- | --- | --- | --- | --- |
| Fibrosis risk: LSM >= 8 | 1.00 (0.65–1.54) | 1.11 (0.59–2.09) | 1.17 (0.38–3.64) | 1.12 (0.69–1.82) | 0.95 (0.58–1.54) |
| - BMI | 1.14 (1.11–1.18)*** | 1.12 (1.08–1.17)*** | 1.13 (1.08–1.18)*** | 1.14 (1.10–1.18)*** | 1.15 (1.11–1.19)*** |
| - Age | 1.01 (0.99–1.03) | 1.01 (0.98–1.03) | 1.00 (0.98–1.03) | 1.01 (0.99–1.03) | 1.01 (0.98–1.03) |
| - Sex | 0.48 (0.31–0.75)** | 0.42 (0.25–0.70)** | 0.35 (0.19–0.63)*** | 0.45 (0.28–0.74)** | 0.46 (0.28–0.74)** |
| Fibrosis risk: LSM >= 12 | 1.03 (0.59–1.80) | 1.23 (0.53–2.84) | 1.00 (omitted) | 1.15 (0.62–2.12) | 1.04 (0.55–1.94) |
| - BMI | 1.11 (1.07–1.14)*** | 1.09 (1.04–1.15)*** | 1.10 (1.04–1.16)** | 1.10 (1.06–1.15)*** | 1.11 (1.07–1.15)*** |
| - Age | 1.02 (0.99–1.04) | 1.03 (1.00–1.07) | 1.03 (0.99–1.08) | 1.02 (0.99–1.05) | 1.02 (0.99–1.05) |
| - Sex | 0.71 (0.40–1.25) | 0.78 (0.39–1.55) | 0.62 (0.28–1.35) | 0.73 (0.39–1.36) | 0.68 (0.37–1.26) |
| Fibrosis risk: Agile3+ ≥0.679 | 1.17 (0.71–1.92) | 1.79 (0.85–3.78) | 0.34 (0.04–2.69) | 1.55 (0.91–2.64) | 1.21 (0.69–2.13) |
| - BMI | 1.03 (1.00–1.06)* | 1.04 (0.99–1.08) | 1.03 (0.98–1.09) | 1.03 (0.99–1.06) | 1.04 (1.00–1.07)* |
| - Age | 1.02 (1.00–1.05)* | 1.06 (1.02–1.09)** | 1.06 (1.02–1.10)** | 1.02 (1.00–1.05) | 1.03 (1.01–1.06)** |
| - Sex | 0.89 (0.52–1.51) | 0.98 (0.51–1.86) | 0.83 (0.40–1.72) | 0.88 (0.50–1.55) | 0.83 (0.47–1.47) |
| Cirrhosis risk: Agile4 ≥ 0.565 | 1.31 (0.77–2.23) | 1.86 (0.85–4.03) | 0.41 (0.05–3.20) | 1.82 (1.04–3.21)* | 1.26 (0.69–2.30) |
| - BMI | 1.02 (0.99–1.05) | 1.02 (0.97–1.07) | 0.99 (0.93–1.05) | 1.01 (0.97–1.04) | 1.02 (0.98–1.06) |
| - Age | 1.01 (0.98–1.03) | 1.03 (1.00–1.07)* | 1.03 (0.99–1.07) | 1.00 (0.98–1.03) | 1.01 (0.99–1.04) |
| - Sex | 1.07 (0.60–1.92) | 1.19 (0.58–2.44) | 1.07 (0.46–2.47) | 1.12 (0.60–2.10) | 1.01 (0.53–1.90) |
| Steatosis Risk, CAP ≥ 270 | 1.74 (1.18–2.55)** | 1.79 (0.98–3.26) | 1.19 (0.49–2.91) | 1.61 (1.02–2.53)* | 1.76 (1.14–2.72)* |
| - BMI | 1.14 (1.10–1.18) | 1.16 (1.11–1.21) | 1.14 (1.09–1.20)*** | 1.16 (1.11–1.20)*** | 1.14 (1.10–1.18)*** |
| - Age | 1.02 (1.00–1.04) | 1.03 (1.00–1.05) | 1.03 (1.01–1.05) | 1.03 (1.01–1.05) | 1.02 (1.00–1.04) |
| - Sex | 0.26 (0.16–0.42) | 0.21 (0.12–0.38) | 0.18 (0.09–0.35) | 0.26 (0.15–0.45) | 0.21 (0.12–0.37) |

Odds of Liver Health Outcomes (binomial logistic regression) among participants with and without mental disorders, adjusted for BMI, age, and sex Significant associations (p < 0.05,0.01,0.001) are marked with an asterisk (*,**,***). Abbreviations: LSM = Liver Stiffness Measurement, Agile = Age, Gender, Insulin Resistance, and Liver Fibrosis Score, CAP = Controlled Attenuation Parameter.

**Supplementary Table S3F** Model details Sleep Apnea Outcomes

|  | **Any MD**  **OR (95% CI)** | **SCH**  **OR (95% CI)** | **BD**  **OR (95% CI)** | **DEP**  **OR (CI95%)** | **ANX**  **OR (95% CI)** |
| --- | --- | --- | --- | --- | --- |
| Any OSA, AHI ≥ 15 or CPAP treated | 1.00 (0.66–1.53) | 1.31 (0.65–2.64) | 2.01 (0.72–5.59) | 0.97 (0.60–1.56) | 1.18 (0.73–1.92) |
| - BMI | 1.08 (1.05–1.12)*** | 1.10 (1.05–1.15)*** | 1.10 (1.05–1.16)*** | 1.08 (1.04–1.12)*** | 1.09 (1.05–1.12)*** |
| - Age | 1.09 (1.06–1.11)*** | 1.10 (1.07–1.14)*** | 1.11 (1.07–1.14)*** | 1.09 (1.06–1.12)*** | 1.10 (1.07–1.13)*** |
| - Sex | 0.28 (0.17–0.45)*** | 0.26 (0.14–0.48)*** | 0.25 (0.13–0.51)*** | 0.25 (0.14–0.42)*** | 0.23 (0.13–0.39)*** |
| Severe OSA AHI ≥ 30 | 0.68 (0.36–1.26) | 1.22 (0.46–3.20) | 0.88 (0.18–4.28) | 0.62 (0.31–1.26) | 1.00 (0.50–1.98) |
| - BMI | 1.05 (1.01–1.09)* | 1.06 (1.01–1.12)* | 1.07 (1.01–1.14)* | 1.05 (1.01–1.10)* | 1.05 (1.01–1.09)* |
| - Age | 1.04 (1.01–1.07)** | 1.05 (1.01–1.09)** | 1.04 (1.00–1.08)* | 1.03 (1.00–1.06)* | 1.05 (1.02–1.08)** |
| - Sex | 0.16 (0.09–0.30)*** | 0.14 (0.07–0.28)*** | 0.15 (0.07–0.33)*** | 0.22 (0.12–0.42)*** | 0.16 (0.08–0.30)*** |

Odds of Sleep Apnea Outcomes (binomial logistic regression) among participants with and without mental disorders, adjusted for BMI, age, and sex. Significant associations (p < 0.05,0.01,0.001) are marked with an asterisk (*,**,***). Abbreviations: OSA = Obstructive Sleep Apnea, AHI = Apnea-Hypopnea Index, CPAP = Continuous Positive Airway Pressure.

**Supplementary Table S4 – Odds of somatic disease stratified by sex adjusted for age and BMI**

| **Category** | **Outcome** | **AnyMD Men (n=83)**  **OR (95% CI)** | **AnyMD Women (n=262)**  **OR (95% CI)** |
| --- | --- | --- | --- |
| Glucometabolic parameters | IFG | 1.05 (0.54–2.02) | 1.31 (0.82–2.09) |
|  | Prediabetes | 2.03 (0.25–16.29) | 0.55 (0.20–1.54) |
|  | Diabetes | 1.11 (0.46–2.71) | 1.28 (0.68–2.40) |
|  | HOMA2-IR > 1.4 | 0.08 (0.00–2.17) | 1.27 (0.68–2.37) |
| Metabolic Syndrome | MetS | 1.22 (0.53–2.80) | 1.65 (1.08–2.50) |
| Blood Lipid Abnormalities | LDL-C ≥ 3 mmol/l | 1.73 (0.91–3.26) | 0.71 (0.48–1.05) |
|  | Triglycerides ≥ 1.7 mmol/l | 1.67 (0.90–3.11) | 2.10 (1.39–3.18)*** |
|  | HDL-C < 1.3 (M) or < 1.0 (FM) | 1.05 (0.56–1.97) | 1.18 (0.79–1.75) |
|  | Total-C/HDL Ratio > 6.0 | 2.91 (1.33–6.33)** | 1.26 (0.64–2.51) |
|  | HDL-Triglycerides ratio ≥ 2.97 (M) or ≥ 2.23 (FM) | 2.35 (1.11–5.00)* | 2.64 (1.43–4.87)** |
| Blood Pressure Outcomes | Hypertension | 0.71 (0.35–1.43) | 0.88 (0.59–1.31) |
|  | Hypotension | 0.57 (0.23–1.44) | 1.11 (0.74–1.68) |
|  | Reverse Dipper > 0% BP dip | 0.39 (0.10–1.58) | 1.68 (0.78–3.61) |
|  | Extreme Dipper > 20% BP dip | 0.75 (0.25–2.25) | 0.46 (0.23–0.91) ∗ |
|  | Non-Dipper < 10% BP dip | 1.82 (0.77–4.34) | 1.49 (0.85–2.62) |
| Liver Health Outcomes | Fibrosis risk: LSM ≥ 8 | 0.66 (0.29–1.49) | 1.28 (0.75–2.18) |
|  | Fibrosis risk: LSM ≥ 12 | 0.91 (0.30–2.74) | 1.16 (0.59–2.29) |
|  | Fibrosis risk: Agile3+ ≥ 0.679 | 1.17 (0.44–3.12) | 1.22 (0.68–2.21) |
|  | Cirrhosis risk: Agile4 ≥ 0.565 | 1.48 (0.51–4.25) | 1.29 (0.69–2.41) |
|  | Steatosis Risk, CAP ≥ 270 | 0.94 (0.37–2.40) | 1.94 (1.28–2.94)** |
| Sleep Apnea Outcomes | Any OSA, AHI ≥ 15 or CPAP treated | 0.95 (0.41–2.19) | 1.00 (0.61–1.64) |
|  | Severe OSA AHI ≥ 30 | 0.56 (0.21–1.46) | 0.74 (0.32–1.69) |

Odds of obesity-associated disease outcomes (binomial logistic regression) among participants with mental disorder compared to participant without mental disorders, stratified by sex and adjusted for BMI and age. Significant associations (p < 0.05, 0.01, 0.001) are marked with an asterisk (*, **, ***). Abbreviations: AnyMD = Any Mental Disorder; IFG = Impaired Fasting Glucose; HOMA2-IR = Homeostasis Model Assessment of Insulin Resistance; MetS = Metabolic Syndrome; LDL-C = Low-Density Lipoprotein Cholesterol; HDL-C = High-Density Lipoprotein Cholesterol; BP = Blood Pressure; LSM = Liver Stiffness Measurement; CAP = Controlled Attenuation Parameter; AHI = Apnea-Hypopnea Index; CPAP = Continuous Positive Airway Pressure.

# Visual illustration of BMI’s association with obesity associated disease

As shown in Tables S3A–S3F, BMI is a significant cofactor influencing the risk of most obesity-associated diseases. To visualize this relationship in greater detail, we derived marginal effects of BMI from models predicting obesity-associated diseases, adjusting for the covariates AnyMD, BMI, age, and sex (as presented in the first result column of Tables 3A–3F). Predicted probabilities were calculated for BMI values ranging from 30 to 60, in increments of 3, offering a visual representation of the associations between BMI and health outcomes adjusted for age, sex and mental disease status (Figure S1).


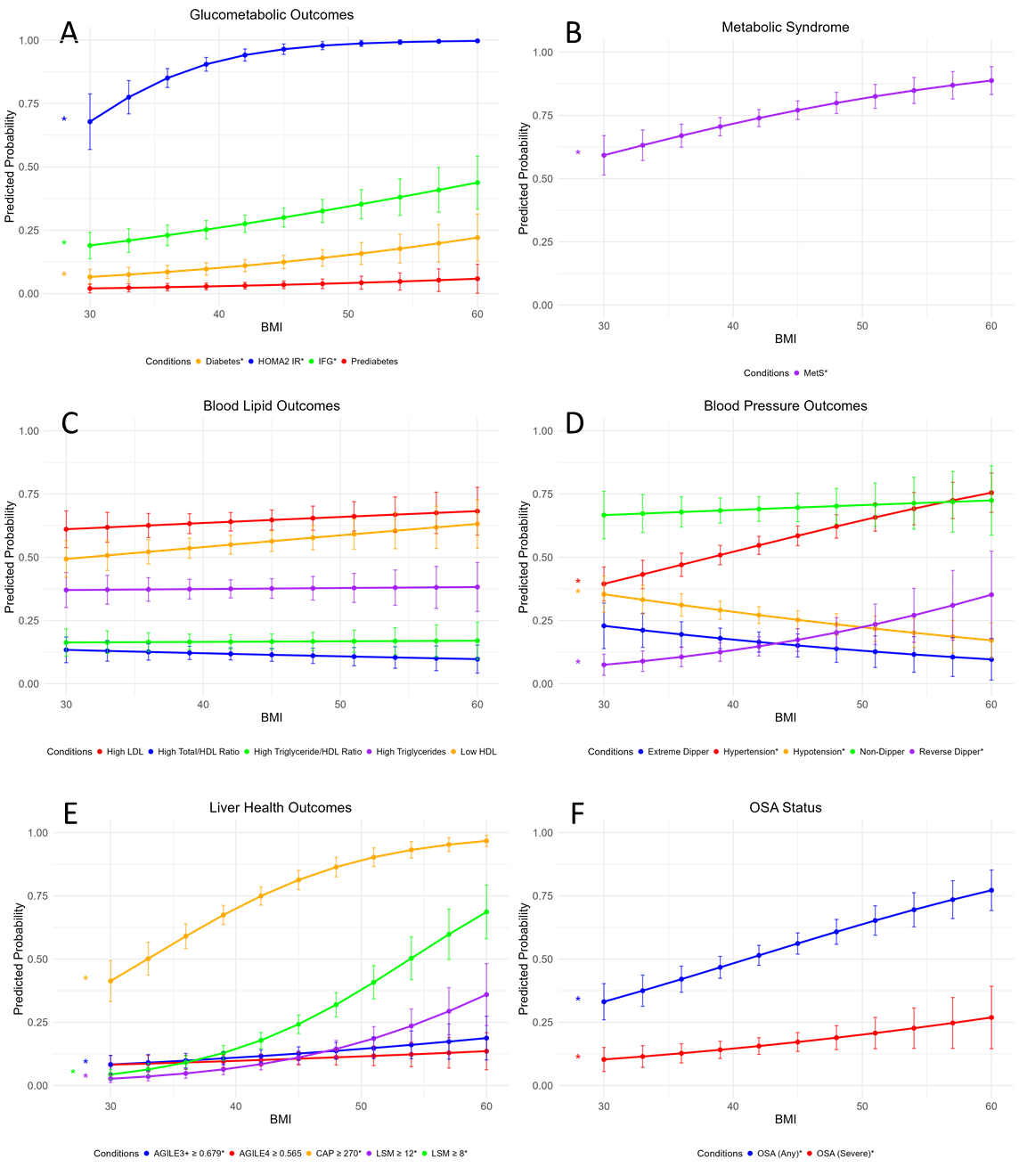


**Figure S2**. *The relative contribution of BMI on obesity associated health outcomes*. Predicted probabilities of obesity-associated health outcomes across BMI levels. Estimates are derived from logistic regression models adjusted for age, sex, and mental disorder status (AnyMD). * indicates BMI was a significant covariate in the model. (A) Glucometabolic Outcomes: Probability of diabetes, HOMA-IR > 2, impaired fasting glucose (IFG), and prediabetes. (B) Metabolic Syndrome: Probability of metabolic syndrome (MetS). (C) Blood Lipid Outcomes: Probability of lipid abnormalities, including high LDL, high total cholesterol-HDL ratio, high triglyceride-HDL ratio, high triglycerides, and low HDL. (D) Blood Pressure Outcomes: hypertension, hypotension extreme dipper, non-, extreme and reverse nocturnal bloodpressure dipping. (E) Liver Health Outcomes: Liver-related markers (Agile4 ≥ 0.565, CAP ≥ 270, LSM ≥ 12, and LSM ≥ 8). (F) Obstructive Sleep Apnea (OSA) Status: Probability of mild and severe OSA. Error bars represent 95% confidence intervals.

# Somatic disease risk adjusted for psychotropics

**Table S5.** Adjusted Odds Ratios of Obesity Associated Disease Outcomes for Users of Psychotropics Compared to Participants Without Mental Disorders and No Psychotropic Use

|  | Any psychotropics (n=235)  (OR (95% CI) | Anxiolytics  (n=11)  OR (95% CI) | Hypnotics  (n=19)  OR (95% CI) | Anti-depressants (n=147)  OR (95% CI) | Mood Stabilizers (n=68)  (OR 95% CI) | Anti-psychotics (n=132)  OR (95% CI) |
| --- | --- | --- | --- | --- | --- | --- |
| **Glucometabolic Health** |  |  |  |  |  |  |
| IFG | 1.2 (0.8–1.9) | 1.0 (0.2–4.3) | 1.5 (0.5–4.2) | 1.6 (1.0–2.5) | 0.9 (0.5–1.8) | 1.5 (0.9–2.5) |
| Prediabetes | 0.7 (0.3–2.1) | 2.1 (0.2–19.2) | 1.0 (0.1–8.6) | 0.7 (0.2–2.3) | 0.8 (0.2–3.6) | 0.6 (0.2–2.5) |
| Diabetes | 1.3 (0.8–2.4) | **4.8 (1.3–18.1)*** | 1.9 (0.6–6.3) | 1.2 (0.6–2.4) | 1.1 (0.5–2.7) | 1.4 (0.7–2.7) |
| HOMA2-IR > 1.4 | 1.3 (0.6–2.7) | 0.0 (omitted) | 1.1 (0.1–9.6) | 1.8 (0.7–4.8) | 0.8 (0.3–2.0) | 0.8 (0.3–2.1) |
| **Metabolic Syndrome** |  |  |  |  |  |  |
| ≥3 MetS Criteria | **1.7 (1.1–2.5)*** | 1 (omitted) | 2.8 (0.6–13.4) | **2.1 (1.2–3.5)*** | 1.4 (0.8–2.7) | 1.6 (1.0–2.7) |
| **Blood Lipids** |  |  |  |  |  |  |
| LDL-C ≥ 3 mmol/l | 0.9 (0.6–1.2) | 0.6 (0.2–1.9) | 0.5 (0.2–1.4) | 0.8 (0.6–1.3) | 1.0 (0.6–1.9) | 0.9 (0.6–1.5) |
| TriGly ≥ 1.7 mmol/l | **2.0 (1.4–3.0)*** | 2.2 (0.6–7.6) | 2.4 (0.9–6.5) | **2.6 (1.7–4.1)*** | 1.7 (1.0–3.0) | **1.9 (1.2–3.0)*** |
| HDL < 1.3 (M) or <1.0 (FM) | 1.3 (0.9–1.9) | 1.49 (0.55–4.07) | 1.5 (0.6–4.1) | 1.5 (1.0–2.3) | 1.2 (0.7–2.2) | 1.5 (1.0–2.4) |
| Total-C/HDL Ratio > 6.0 | **2.3 (1.3–3.9)*** | 1.0 (omitted) | 0.6 (0.1–5.0) | **2.8 (1.5–5.1)*** | 1.3 (0.5–3.1) | **2.2 (1.2–4.1)*** |
| Triglycerides-HDL-C ratio ≥ 2.97 (M) or ≥ 2.23 (FM) | **3.0 (1.8–4.9)*** | 3.5 (0.9–14.5) | **4.7 (1.6–13.8)*** | **3.2 (1.8–5.7)*** | **2.9 (1.4–5.9)*** | **3.3 (1.9–5.9)*** |
| **Blood Pressure regulation** |  |  |  |  |  |  |
| Hypertension | 0.8 (0.5–1.1) | 3.2 (0.6–16.5) | 0.8 (0.3–2.2) | 0.9 (0.6–1.4) | **0.5 (0.3–0.9)*** | 0.6 (0.4–1.0) |
| Hypotension | 0.9 (0.6–1.4) | 1 (omitted) | 0.7 (0.2–2.5) | 0.8 (0.5–1.3) | 1.3 (0.7–2.4) | 1.1 (0.7–1.7) |
| Reverse Dipper >0% BP dip | 1.1 (0.5–2.2) | 0.8 (0.1–8.4) | 0.6 (0.1–6.0) | 1.0 (0.5–2.2) | 1.1 (0.4–3.3) | 1.3 (0.6–3.2) |
| Extreme Dipper >20% BP dip | 0.6 (0.3–1.2) | 0.5 (0.1–4.8) | 0.5 (0.1–4.2) | 0.5 (0.3–1.2) | 1.1 (0.4–2.9) | 0.7 (0.3–1.5) |
| Non-Dipper <10% BP dip | 1.4 (0.8–2.3) | 3.1 (0.4–27.6) | 3.8 (0.4–32.6) | 1.7 (0.9–3.1) | 0.6 (0.3–1.2) | 1.5 (0.8–3.0) |
| **Liver Health** |  |  |  |  |  |  |
| Fibrosis risk: LSM >= 8 | 1.2 (0.8–2.0) | 1.4 (0.3–5.8) | 1.2 (0.4–3.9) | 1.1 (0.7–2.0) | 1.2 (0.6–2.5) | 1.4 (0.8–2.4) |
| Fibrosis risk: LSM >= 12 | 1.3 (0.7–2.3) | 2.0 (0.4–10.4) | 2.2 (0.6–7.7) | 1.1 (0.5–2.2) | 1.2 (0.5–3.0) | 1.3 (0.7–2.7) |
| Fibrosis risk: Agile3+ ≥0.679 | 1.4 (0.8–2.4) | 1.8 (0.4–8.8) | 0.4 (0.1–3.1) | 1.5 (0.8–2.8) | 1.2 (0.5–2.7) | 1.3 (0.7–2.6) |
| Cirrhosis risk: Agile4 ≥ 0.565 | 1.5 (0.9–2.7) | 2.3 (0.5–11.4) | 0.6 (0.1–4.5) | 1.8 (1.0–3.4) | 1.2 (0.5–3.0) | 1.3 (0.6–2.7) |
| Steatosis Risk, CAP ≥ 270 | **1.6 (1.1–2.4)*** | 1.1 (0.2–5.0) | 0.7 (0.2–2.1) | **1.8 (1.1–3.0)*** | 1.0 (0.6–1.9) | 1.2 (0.7–2.0) |
| **Sleep Apnea status** |  |  |  |  |  |  |
| Any OSA, AHI ≥ 15 or CPAP treated | 1.1 (0.7–1.8) | 1.2 (0.2–7.1) | 0.9 (0.2–3.4) | 1.4 (0.8–2.4) | 1.5 (0.7–3.1) | 1.2 (0.7–2.2) |
| Severe OSA, AHI ≥ 30 | 0.8 (0.4–1.6) | 1 (omitted) | 0.8 (0.1–8.0) | 1.1 (0.5–2.3) | 0.9 (0.3–2.7) | 0.6 (0.2–1.6) |

Odds of obesity-associated disease outcomes (binomial logistic regression) among users of psychotropic medications compared to participants without mental disorders and no psychotropic use, adjusted for BMI, age, and sex. Significant associations (p < 0.05, chi-square test) are marked with an asterisk (*). Abbreviations: IFG = Impaired Fasting Glucose; HOMA2-IR = Homeostasis Model Assessment of Insulin Resistance; MetS = Metabolic Syndrome; LDL-C = Low-Density Lipoprotein Cholesterol; HDL = High-Density Lipoprotein Cholesterol; BP = Blood Pressure; LSM = Liver Stiffness Measurement; CAP = Controlled Attenuation Parameter; AHI = Apnea-Hypopnea Index; CPAP = Continuous Positive Airway Pressure.

**Table S6.** Logistic Regression Results for Somatic Disease Outcomes Based on Mental Disorders and Psychotropic Medications

| Models without Medication | Models with Antidepressant | Models with Antipsychotics | Models with Mood Stabilizers | Models with Hypnotics | Models with all medications |
| --- | --- | --- | --- | --- | --- |
| Mets | & Antidepressants | & Antipsychotics | & Mood Stabilizers | & Hypnotics | & All Medications |
| **BMI: OR 1.1 (1.0-1.1) *****  **Age: OR 1.0 (1.0-1.1) *****  **Sex: OR 0.4 (0.3-0.7) *****  **AnyMD: OR 1.6 (1.1-2.3) *** | **BMI: OR 1.1 (1.0-1.1) *****  **Age: OR 1.0 (1.0-1.1) *****  **Sex: OR 0.4 (0.3-0.7) *****  **AnyMD: OR 1.3 (0.8-2.0)**  **Med1: OR 1.6 (0.9-2.8)** | BMI: OR 1.1 (1.0-1.1) ***  Age: OR 1.0 (1.0-1.1) ***  Sex: OR 0.4 (0.3-0.7) ***  AnyMD: OR 1.5 (1.0-2.4) *  Med2: OR 1.0 (0.6-1.7) | BMI: OR 1.1 (1.0-1.1) ***  Age: OR 1.0 (1.0-1.1) ***  Sex: OR 0.4 (0.3-0.7) ***  AnyMD: OR 1.6 (1.1-2.4) *  Med3: OR 0.9 (0.5-1.7) | BMI: OR 1.1 (1.0-1.1) ***  Age: OR 1.0 (1.0-1.1) ***  Sex: OR 0.4 (0.3-0.7) ***  AnyMD: OR 1.5 (1.0-2.2) *  Med4: OR 2.0 (0.4-9.1) | BMI: OR 1.1 (1.0-1.1) ***  Age: OR 1.0 (1.0-1.1) ***  Sex: OR 0.4 (0.3-0.7) ***  AnyMD: OR 1.3 (0.8-2.1)  Med1: OR 1.6 (0.9-2.8)  Med2: OR 1.0 (0.6-1.7)  Med3: OR 0.8 (0.4-1.6)  Med4: OR 1.8 (0.4-8.5) |
| High Triglycerides | & Antidepressants | & Antipsychotics | & Mood Stabilizers | & Hypnotics | & All Medications |
| **BMI: OR 1.0 (1.0-1.0)**  **Age: OR 1.0 (1.0-1.0) ***  **Sex: OR 0.5 (0.3-0.7) *****  **AnyMD: OR 2.0 (1.4-2.8) ***** | BMI: OR 1.0 (1.0-1.0)  Age: OR 1.0 (1.0-1.0) *  Sex: OR 0.5 (0.3-0.7) ***  AnyMD: OR 1.6 (1.1-2.4) *  Med1: OR 1.7 (1.1-2.6) * | **BMI: OR 1.0 (1.0-1.0)**  **Age: OR 1.0 (1.0-1.0) ***  **Sex: OR 0.5 (0.3-0.7) *****  **AnyMD: OR 2.0 (1.4-3.0) *****  **Med2: OR 0.9 (0.6-1.5)** | BMI: OR 1.0 (1.0-1.0)  Age: OR 1.0 (1.0-1.0) *  Sex: OR 0.5 (0.3-0.7) ***  AnyMD: OR 2.1 (1.5-3.0) ***  Med3: OR 0.8 (0.4-1.4) | BMI: OR 1.0 (1.0-1.0)  Age: OR 1.0 (1.0-1.0) *  Sex: OR 0.5 (0.3-0.7) ***  AnyMD: OR 2.0 (1.4-2.8) ***  Med4: OR 1.2 (0.5-3.1) | BMI: OR 1.0 (1.0-1.0)  Age: OR 1.0 (1.0-1.0) *  Sex: OR 0.5 (0.3-0.7) ***  AnyMD: OR 1.7 (1.1-2.6) *  Med1: OR 1.7 (1.1-2.7) *  Med2: OR 0.9 (0.6-1.5)  Med3: OR 0.7 (0.4-1.3)  Med4: OR 1.1 (0.4-3.1) |
| High Total-C-HDL ratio | & Antidepressants | & Antipsychotics | & Mood Stabilizers | & Hypnotics | & All Medications |
| **BMI: OR 1.0 (1.0-1.0)**  **Age: OR 1.0 (1.0-1.0)**  **Sex: OR 0.3 (0.2-0.5) *****  **AnyMD: OR 1.9 (1.1-3.1) *** | **BMI: OR 1.0 (1.0-1.0)**  **Age: OR 1.0 (1.0-1.0)**  **Sex: OR 0.3 (0.2-0.5) *****  **AnyMD: OR 1.3 (0.7-2.5)**  **Med1: OR 2.0 (1.1-3.8) *** | BMI: OR 1.0 (1.0-1.0)  Age: OR 1.0 (1.0-1.0)  Sex: OR 0.3 (0.2-0.6) ***  AnyMD: OR 1.6 (0.9-2.9)  Med2: OR 1.4 (0.7-2.6) | BMI: OR 1.0 (1.0-1.0)  Age: OR 1.0 (1.0-1.0)  Sex: OR 0.3 (0.2-0.5) ***  AnyMD: OR 2.0 (1.2-3.4) *  Med3: OR 0.7 (0.3-1.6) | BMI: OR 1.0 (1.0-1.0)  Age: OR 1.0 (1.0-1.0)  Sex: OR 0.3 (0.2-0.5) ***  AnyMD: OR 1.9 (1.2-3.3) *  Med4: OR 0.4 (0.05-2.8) | BMI: OR 1.0 (1.0-1.0)  Age: OR 1.0 (1.0-1.0)  Sex: OR 0.3 (0.2-0.6) ***  AnyMD: OR 1.3 (0.6-2.5)  Med1: OR 2.1 (1.1-4.0) *  Med2: OR 1.5 (0.8-2.9)  Med3: OR 0.6 (0.2-1.4)  Med4: OR 0.3 (0.04-2.5) |
| High Triglycerides -HDL ratio | & Antidepressants | & Antipsychotics | & Mood Stabilizers | & Hypnotics | & All Medications |
| **BMI: OR 1.0 (1.0-1.0)**  **Age: OR 1.0 (1.0-1.0)**  **Sex: OR 0.5 (0.3-0.8) ****  **AnyMD: OR 2.5 (1.6-4.1)** | **BMI: OR 1.0 (1.0-1.0)**  **Age: OR 1.0 (1.0-1.0)**  **Sex: OR 0.5 (0.3-0.8) ****  **AnyMD: OR 2.3 (1.3-3.8) ****  **Med1: OR 1.3 (0.8-2.2)** | **BMI: OR 1.0 (1.0-1.0)**  **Age: OR 1.0 (1.0-1.0)**  **Sex: OR 0.5 (0.3-0.8) ****  **AnyMD: OR 2.3 (1.3-3.9) ****  **Med2: OR 1.3 (0.8-2.2)** | **BMI: OR 1.0 (1.0-1.0)**  **Age: OR 1.0 (1.0-1.0)**  **Sex: OR 0.5 (0.3-0.8) ****  **AnyMD: OR 2.5 (1.5-4.1) *****  **Med3: OR 1.1 (0.6-2.1)** | **BMI: OR 1.0 (1.0-1.0)**  **Age: OR 1.0 (1.0-1.0)**  **Sex: OR 0.5 (0.3-0.8) ****  **AnyMD: OR 2.4 (1.5-3.9) *****  **Med4: OR 2.0 (0.7-5.6)** | **BMI: OR 1.0 (1.0-1.0)**  **Age: OR 1.0 (1.0-1.0)**  **Sex: OR 0.5 (0.3-0.8) ****  **AnyMD: OR 2.0 (1.2-3.6) ***  **Med1: OR 1.2 (0.7-2.1)**  **Med2: OR 1.3 (0.7-2.2)**  **Med3: OR 0.9 (0.5-1.9)**  **Med4: OR 1.8 (0.6-5.3)** |
| Extreme-dipping night BP | & Antidepressants | & Antipsychotics | & Mood Stabilizers | & Hypnotics | & All Medications |
| **BMI: OR 1.0 (0.9-1.0)**  **Age: OR 1.0 (0.9-1.0) ****  **Sex: OR 1.1 (0.6-2.0)**  **AnyMD: OR 0.5 (0.3-0.9) *** | **BMI: OR 1.0 (0.9-1.0)**  **Age: OR 1.0 (0.9-1.0) ****  **Sex: OR 1.1 (0.6-2.0)**  **AnyMD: OR 0.5 (0.2-1.0) ***  **Med1: OR 1.2 (0.5-2.9)** | **BMI: OR 1.0 (0.9-1.0)**  **Age: OR 1.0 (0.9-1.0) ****  **Sex: OR 1.1 (0.6-2.1)**  **AnyMD: OR 0.4 (0.2-0.8) ***  **Med2: OR 1.8 (0.7-4.3)** | BMI: OR 1.0 (0.9-1.0)  Age: OR 1.0 (0.9-1.0) **  Sex: OR 1.1 (0.6-2.1)  AnyMD: OR 0.4 (0.2-0.8) **  Med3: OR 2.8 (1.0-7.7) * | BMI: OR 1.0 (0.9-1.0)  Age: OR 1.0 (0.9-1.0) **  Sex: OR 1.1 (0.6-2.0)  AnyMD: OR 0.5 (0.3-0.9) *  Med4: OR 1.1 (0.1-9.7) | **BMI: OR 1.0 (0.9-1.0)**  **Age: OR 1.0 (0.9-1.0) ****  **Sex: OR 1.1 (0.6-2.1)**  **AnyMD: OR 0.4 (0.2-0.8) ***  **Med1: OR 1.1 (0.4-2.6)**  **Med2: OR 1.5 (0.6-3.8)**  **Med3: OR 2.5 (0.9-7.3)**  **Med4: OR 0.8 (0.1-7.4)** |
| Steatosis (CAP ≥ 270) | & Antidepressants | & Antipsychotics | & Mood Stabilizers | & Hypnotics | & All Medications |
| **BMI: OR 1.1 (1.1-1.2) *****  **Age: OR 1.0 (1.0-1.0) ***  **Sex: OR 0.3 (0.2-0.4) *****  **AnyMD: OR 1.7 (1.2-2.6) **** | **BMI: OR 1.1 (1.1-1.2) *****  **Age: OR 1.0 (1.0-1.0) ***  **Sex: OR 0.3 (0.2-0.4) *****  **AnyMD: OR 1.8 (1.1-2.8) ***  **Med1: OR 1.0 (0.6-1.7)** | BMI: OR 1.1 (1.1-1.2) ***  Age: OR 1.0 (1.0-1.0) *  Sex: OR 0.2 (0.1-0.4) ***  AnyMD: OR 2.2 (1.4-3.4) ***  Med2: OR 0.6 (0.3-1.0) * | BMI: OR 1.1 (1.1-1.2) ***  Age: OR 1.0 (1.0-1.0) *  Sex: OR 0.3 (0.2-0.4) ***  AnyMD: OR 2.1 (1.4-3.1) ***  Med3: OR 0.5 (0.3-0.9) * | BMI: OR 1.1 (1.1-1.2) ***  Age: OR 1.0 (1.0-1.0) *  Sex: OR 0.3 (0.2-0.4) ***  AnyMD: OR 1.8 (1.2-2.7) **  Med4: OR 0.4 (0.1-1.2) | BMI: OR 1.1 (1.1-1.2) ***  Age: OR 1.0 (1.0-1.0) *  Sex: OR 0.2 (0.1-0.4) ***  AnyMD: OR 2.4 (1.4-3.9) ***  Med1: OR 1.1 (0.6-1.9)  Med2: OR 0.7 (0.4-1.2)  Med3: OR 0.6 (0.3-1.1)  Med4: OR 0.5 (0.1-1.5) |

This table presents the odds ratios and confidence intervals for the association between various mental disorder groups and the presence of somatic health outcomes, under different medication models. The comparison group are, in all cases, participants without mental disorders not using psychotropics. Medication (Med1 - Med4): Represents classes of psychotropic drugs (Antidepressants, Antipsychotics, Mood Stabilizers, Hypnotics, respectively) as detailed in their respective columns. Rows detail variables in each model and below variable effects. *,** and *** denotes significant effects at p> 0.05, 0.01 and 0.001, respectively. Dependent variables are in all cases the first listed variabel in each model.

# Post-hoc test: Controlling for multiplicity - Benjamini-Hochberg procedure

To control for multiple comparisons, we applied the Benjamini-Hochberg (BH) procedure to the p-values from the binominal regression models assessing the odds of all disease outcomes for people with mental disorders (AnyMD) compared to people without mental disorders (NoMD) adjusted for BMI, age and sex. These are the model results displayed in result column 1, supplementary table S3A-S3F

The BH procedure adjusts the false discovery rate, controlling for the expected proportion of false positives among the significant results.

We ranked the p-values from smallest to largest, assigning each p-value a rank. Next, we calculated an adjusted threshold for significance by multiplying the rank of each p-value by the total number of tests and the chosen false discovery rate level of 0.05. This approach allowed us to set an increasing threshold for significance as the rank of each p-value increased. We regarded outcomes as significant if their p-value was less than or equal to the calculated threshold.

The results of this procedure are shown below:

**Table S7.** Model results adjusted for False Discovery Rate

| **Outcome** | **P-Value** | **Rank** | **FDR Threshold** | **Significant** |
| --- | --- | --- | --- | --- |
| TriGly ≥ 1.7 mmol/l | 0.0000776302 | 1 | 0.0022727273 | **True** |
| TriGly-HDL-C ratio | 0.0001041480 | 2 | 0.0045454545 | **True** |
| Steatosis risk (CAP ≥ 270) | 0.0047118616 | 3 | 0.0068181818 | **True** |
| Total-C/HDL Ratio > 6.0 | 0.0175142379 | 4 | 0.0090909091 | False |
| ≥3 MetS Criteria | 0.0212919709 | 5 | 0.0113636364 | False |
| Extreme Dipper >20% BP dip | 0.0281897273 | 6 | 0.0136363636 | False |
| Non-Dipper <10% BP dip | 0.0541320119 | 7 | 0.0159090909 | False |
| Diabetes | 0.4371292330 | 8 | 0.0181818182 | False |
| Prediabetes | 0.4639780552 | 9 | 0.0204545455 | False |
| Impaired Fasting Glucose | 0.3067837545 | 10 | 0.0227272727 | False |
| HDL-C < 1.3 (M) or <1.0 (FM) | 0.3788717817 | 11 | 0.0250000000 | False |
| Hypertension | 0.3194648033 | 12 | 0.0272727273 | False |
| Hypotension | 0.9313397379 | 13 | 0.0295454545 | False |
| Reverse Dipper >0% BP dip | 0.7225145913 | 14 | 0.0318181818 | False |
| Fibrosis risk: LSM ≥ 8 | 0.9846769795 | 15 | 0.0340909091 | False |
| Fibrosis risk: LSM ≥ 12 | 0.9259859197 | 16 | 0.0363636364 | False |
| Fibrosis risk: Agile3+ ≥0.679 | 0.5449773281 | 17 | 0.0386363636 | False |
| Cirrhosis risk: Agile4 ≥ 0.565 | 0.3228390263 | 18 | 0.0409090909 | False |
| Any OSA, AHI ≥ 15 or CPAP treated | 0.2179370890 | 19 | 0.0431818182 | False |
| Severe OSA AHI ≥ 30 | 0.9894056475 | 20 | 0.0454545455 | False |
| LDL-C ≥ 3 mmol/l | 0.5762073280 | 21 | 0.0477272727 | False |
| HOMA2-IR > 1.4 | 0.7098529364 | 22 | 0.0500000000 | False |

Three outcomes remained significant after applying the BH-adjusted FDR threshold:

1. **Elevated Triglycerides** (p = 0.0000776302)
2. **Elevated Triglycerides-HDL-ratio** (p = 0.0001041480)
3. **Steatosis Risk (CAP ≥ 270)** (p = 0.0047118616)

No other disease outcomes showed significantly higher odds in individuals with mental disorders compared to those without, after accounting for multiple comparisons.

# Baseline characteristics of excluded participants

To evaluate whether exclusion of 261 participants from the primary analyses might have introduced bias, we compared their baseline characteristics (Table S1A) and health outcome prevalence (Table S1B) with those of the two main study groups: participants without mental disorders (NoMD) and participants with any mental disorder (AnyMD). Across most measures, the excluded group resembled the NoMD group more closely, with values generally falling between those of NoMD and AnyMD. No consistent pattern emerged to suggest systematic bias from these exclusions.

**Table S8A:** Baseline charectaristics of included and excluded partipants

| **Participant characteristics** | **No MD (n=317)** | **Any MD (n=345)** | **Excluded (n=261)** | **ANOVA p-value** |
| --- | --- | --- | --- | --- |
| **Age (years)** | 44.1 ± 11.1 ^B^ | 39.3 ± 10.9 ^A,C^ | 42.6 ± 11.4 ^B^ | < 0.001 |
| **BMI (kg/m²)** | 40.9 ± 6.5 ^B^ | 42.8 ± 7.9 A, ^A,C^ | 40.9 ± 6.3 ^B^ | < 0.001 |
| **Hip:Waist ratio** | 1.06 ± 0.12 | 1.07 ± 0.11 | 1.08 ± 0.12 | 0.129 |
| **Body Fat (%)** | 46.3 ± 6.8 ^B^ | 48.1 ± 6.0 A, ^A,C^ | 46.6 ± 6.4 ^B^ | < 0.001 |
| **VAT–SAT ratio** | 0.44 ± 0.28 ^B, C^ | 0.39 ± 0.24 ^A^ | 0.39 ± 0.25 ^A^ | 0.026 |
| **Categorical Variable** |  |  |  | ***χ*² p-value** |
| **Sex (Female)** | 67.2% ^B,C^ | 78.6% ^A^ | 72.0% ^A^ | 0.004 |
| **Currently working (Yes)** | 85.3% ^B,C^ | 38.9% ^A,C^ | 66.3% ^A,B^ | <0.001 |
| **Anxiolytics** | 0.0% ^B^ | 3.2% ^A,C^ | 0.8% ^B^ | 0.001 |
| **Hypnotics** | 0.0% ^B,C^ | 5.5% ^A,C^ | 1.5% ^A,B^ | <0.001 |
| **Mood stabilizers** | 0.0% ^B,C^ | 19.7% ^A,C^ | 12.3% ^A,B^ | <0.001 |
| **Antipsychotics** | 0.0% ^B,C^ | 38.3% ^A,C^ | 4.6% ^A,B^ | <0.001 |
| **Antidepressants** | 0.0% ^B,C^ | 42.6% ^A,C^ | 15.7% ^A,B^ | <0.001 |

The table displays participant characteristics across three groups: individuals without mental disorders (NoMD, n=317), individuals with mental disorders (AnyMD, n=345), and participants excluded from the main analysis due to ambiguous or incomplete classification (Excluded, n=261). Continuous variables are presented as mean ± standard deviation, with p-values based on one-way ANOVA across groups. Categorical variables are shown as the percentage of participants with a "yes" response, and p-values are based on Pearson’s chi-squared tests. Superscript letters denote significant pairwise post hoc differences (p < 0.05): A indicates a significant difference from NoMD, B from AnyMD, and C from Excluded.

**Table S8B:** Disease prevalence of partipants included and excluded from analysis

| **Outcome** | **NoMD (%)** | **AnyMD (%)** | **Excluded (%)** | **p-value** |
| --- | --- | --- | --- | --- |
| Impaired fasting glucose | 28.3% | 27.4% | 29.4% | 0.861 |
| Prediabetes | 3.8% | 2.6% | 3.1% | 0.684 |
| Diabetes | 11.4% | 11.3% | 11.5% | 0.997 |
| HOMA2-IR (IR%) | 89.4% | 91.3% | 86.8% | 0.256 |
| MetS | 70.3% | 75.7% | 73.2% | 0.306 |
| High LDL | 64.7% | 63.5% | 64.8% | 0.931 |
| High triglycerides | 31.5% ^B^ | 43.2% ^A^ | 37.2% | 0.008 |
| Low HDL | 49.0% ^B^ | 60.4% ^A^ | 56.1% | 0.014 |
| High Total:HDL ratio | 9.2% ^B^ | 14.2% ^A^ | 8.4% | 0.038 |
| High Trig:HDL ratio | 10.6% ^B^ | 22.1% ^A,C^ | 12.5% ^B^ | 0.000 |
| Hypertension | 59.6% ^B^ | 49.9% ^A,C^ | 58.6% ^B^ | 0.022 |
| Hypotension | 25.6% | 29.3% | 26.8% | 0.563 |
| Non-dipper | 63.4% ^B^ | 74.1% ^A, C^ | 61.4% ^A, B^ | 0.028 |
| Reverse dipper | 13.4% | 15.9% | 8.3% | 0.138 |
| Extreme dipper | 21.5% | 13.2% | 15.9% | 0.106 |
| Fibrosis risk (LSM≥8) | 19.9% | 21.7% | 22.6% | 0.709 |
| Fibrosis risk (LSM≥12) | 9.5% | 10.7% | 9.2% | 0.788 |
| Agile 3+ | 11.4% | 12.5% | 8.8% | 0.357 |
| Agile 4 | 8.8% | 11.3% | 6.9% | 0.171 |
| Steatosis risk | 66.6% ^B^ | 75.9% ^A^ | 73.2% | 0.024 |
| Any OSA | 42.4% | 47.6% | 55.2% | 0.064 |
| Severe OSA | 20.9% ^B^ | 10.7% ^A, C^ | 20.1% ^B^ | 0.010 |

Group-level differences in metabolic, cardiovascular, hepatic, and sleep-related health indicators across participants with no mental disorder (NoMD), any mental disorder (AnyMD), and those excluded from main analyses (Excluded). p-values are based on Pearson’s chi-squared tests comparing the full three-group distribution. Superscript letters indicate statistically significant pairwise differences (A = different from NoMD; B = different from AnyMD; C = different from Excluded), based on follow-up chi-squared tests between pairs of groups. Post hoc comparisons are not corrected for multiple testing.
